# Autophagy induced by Vip3Aa has a pro-survival role in *Spodoptera frugiperda* Sf9 cells

**DOI:** 10.1080/21505594.2021.1878747

**Published:** 2021-01-28

**Authors:** Xiaoyue Hou, Lu Han, Baoju An, Jun Cai

**Affiliations:** a Department of Microbiology, College of Life Sciences, Nankai University, Tianjin, China; b Jiangsu Key Laboratory of Marine Bioresources and Environment, Jiangsu Ocean University, Lianyungang, China; c College of Food Science and Engineering, Jiangsu Ocean University, Lianyungang, China; d Key Laboratory of Molecular Microbiology and Technology, Ministry of Education, Tianjin, China; e Tianjin Key Laboratory of Microbial Functional Genomics, Tianjin, China

**Keywords:** Vip3Aa, autophagy, autophagosome, atg5, sf9 cells

## Abstract

Vip3Aa is an insecticidal protein that can effectively control certain lepidopteran pests and has been used widely in biological control. However, the mechanism of action of Vip3Aa is unclear. In the present study, we showed that Vip3Aa could cause autophagy in Sf9 cells, which was confirmed by the increased numbers of GFP-Atg8 puncta, the appearance of autophagic vacuoles, and an elevated Atg8-II protein level. Moreover, we found that the AMPK-mTOR-ULK1 pathway is involved in Vip3Aa-induced autophagy, which might be associated with the destruction of ATP homeostasis in Vip3Aa-treated cells. Both the elevated p62 level and the increased numbers of GFP-RFP-Atg8 yellow fluorescent spots demonstrated that autophagy in Sf9 cells was inhibited at 24 h after Vip3Aa treatment. With the prolongation of Vip3Aa treatment time, this inhibition became more serious and led to autophagosome accumulation. Genetic knockdown of *ATG5* or the use of the autophagy inhibitor 3-MA further increased the sensitivity of Sf9 cells to Vip3Aa. Overexpression of *ATG5* reduced the cell mortality of Vip3Aa-treated cells. In summary, the results revealed that autophagy induced by Vip3Aa has a pro-survival role, which might be related to the development of insect resistance.

## Introduction

*Bacillus thuringiensis* (Bt) is a microorganism widely used in biological control. Genetically engineered crops expressing Bt protein have been grown commercially for more than two decades to control pests on corn, cotton, and soybean [1]. Bt crops not only control target pests effectively, but also are environmentally friendly and economically beneficial, such as reducing the use of chemical pesticides and crop yield losses [2–4]. Bt insecticidal proteins fall into two broad categories: Insecticidal crystal proteins (ICPs) and Vegetative insecticidal proteins (VIPs). Although industrial preparations of ICPs were used in biological pesticides earlier, most ICPs are not effective in controlling certain agronomically significant lepidopteran, such as *Spodoptera frugiperda* and *Helicoverpa zea*. VIPs have a good control effect on certain lepidopteran pests, and differ from ICPs in their structure, as well as their brush border membrane vesicle binding sites [5,6]. These characteristics of VIPs expand the insecticidal spectrum, and the use of VIPs also delays the insect resistance caused by widespread use of ICPs. Therefore, VIPs are known as the second generation of insecticidal proteins.

According to their amino acid homology, VIPs are divided into four categories: Vip1, Vip2, Vip3, and Vip4. No insects have been identified to sensitive to Vip4; therefore, the other three types of Vip toxins can be divided into two groups according to their mode of action and the object of control: One group comprises binary toxin composed of two elements: Vip1 and Vip2. This binary toxin has strong insecticidal activity against the crucial agricultural insect, *Diabrotica virgifera*, but has no obvious insecticidal activity against lepidopteran insects [7]. The other is Vip3 toxins, which have no sequence similarity with Vip1 or Vip2, and can effectively control Lepidopteran pests, such as *S. frugiperda*.

Currently, most of the reports concerning the mode of action of Vip3 have focused on the Vip3Aa subfamily. According to these studies, two possible mechanisms of Vip3Aa are proposed: Cell death caused by pore formation and apoptotic cell death. Studies have reported that Vip3Aa can form stable ion channels in midgut epithelial cells to achieve its insecticidal effects [5]. When the pH is between 5.0 and 8.0, a current signal could be detected, suggesting that the pore forming ability of Vip3Aa could be regulated by pH [8]. Vip3Aa was reported to cause *S. frugiperda* Sf9 cell apoptosis by activating caspase [9]. Hernandez-Martinez *et al*. performed transcriptome analyses and discovered that Vip3 causes midgut epithelial cell apoptosis in *Spodoptera exigua* larvae [10]. As regards cell apoptosis mechanisms, our previous studies showed that Vip3Aa destroyed the stability of the lysosome membrane, leading to lysosomal pH increase and mitochondrial pathway activation [9,11].

Autophagy affects cell survival by maintaining cell biological functions and removing protein aggregates and damaged organelles. Moreover, several autophagy-related proteins are also involved in exogenous apoptosis pathways, and there is a complex cross-regulation network between autophagy and apoptosis. Autophagy directly or indirectly regulates endogenous mitochondrial pathways. For example, Beclin-1 and Atg4D can be cleaved by caspase protease, while Atg5 can be cleaved by calpain-1 and calpain-2 [12]. These lytic events not only inhibit autophagy, but also enhance cell apoptosis. Bcl-2, which can interact with Beclin-1, is the central regulator of autophagy and apoptosis. Caspase-8 inhibits the interaction between the proteins c-FLIP and Atg3, thereby preventing the interaction between Atg3 and LC3, thus inhibiting autophagy [12]. FADD and Atg5 can directly inhibit cell apoptosis instead of autophagy. Reactive oxygen species (ROS), comprising common cellular stressors, could induce apoptosis. Interestingly, Scherz-Shouval *et al*. found that ROS are essential for autophagy [13]. Therefore, we aimed to study whether Vip3Aa could induce autophagy in Sf9 cells.

Autophagy includes two processes: autophagosomes formation and autophagosomes degradation. Autophagy not only removes misfolded proteins, but also degrades damaged mitochondria or lysosomes to regulate cell apoptosis [14]. In our previous study, we confirmed that Vip3Aa reduced the lysosomal membrane stability in Sf9 cells. Lysosomes are the key organelles for autophagy circulation; therefore, we also aimed to investigate whether autophagy is involved in cell death induced by Vip3Aa.

In the present study, we investigated whether autophagy is related to Vip3Aa-induced cytotoxicity. We found that Vip3Aa can induce autophagy through the AMPK-mTOR-ULK1 pathway in Sf9 cells. Moreover, autophagy could antagonize the virulence of Vip3Aa. The results proved guiding significance and application value to make full use of Vip3Aa, by improving its virulence and delaying insect resistance.

## Materials and methods

### Insect cell culture, antibodies and reagents

*Spodoptera frugiperda* ovarian Sf9 cells were cultivated as described in our previous study [11]. Phosphatase Inhibitor Cocktail (#5870), RIPA buffer (#9806S), and antibodies against AMPK (#2532), phosphorylated (p)-AMPK (Thr172) (#2535), TSC2 (#4308), and p-TSC2 (Ser1387) (#5584) were obtained from Cell Signaling Technology (Beverly, MA, USA). Antibodies against Atg5 (#A19677), Beclin1 (#A7353), p62 (#A7758), LC3-I/II (#A15591), mTOR (#A2445), p-mTOR (Ser2448) (#AP0115), ULK1 (#A8529), p-ULK1 (Ser555) (#AP0760), p-ULK1 (Ser757) (#AP0736), p70S6K (A2190), p-p70S6K (Thr389) (#AP0564), and β-actin (#AC004), and horseradish peroxidase (HRP)-conjugated Goat Anti-Mouse IgG (H + L) (#AS003), and HRP-conjugated Goat Anti-Mouse IgG (H + L) (#AS014) were purchased from ABclonal (Wuhan, China). Cellfectin® II Reagent and PLUS™ Reagent were obtained from Invitrogen (Carlsbad, CA, USA). 3-MA, Rapamycin, and Chloroquine were purchased from Selleck (Houston, TX, USA).

The above antibodies were selected in this study because the mammalian proteins they recognize have a certain homology with the corresponding proteins in *Spodoptera frugiperda*, and the corresponding antigens share common linear epitopes (see the supplementary information). Moreover, in preliminary experiments, using these antibodies, specific protein bands with the expected size had been detected in Sf9 cells.

### Vector construction and Vip3Aa purification

cDNA of Sf9 cells was used as the template for *S. frugiperda ATG8* amplification. The fusion genes, *GFP-ATG8* and *RFP-GFP-ATG8*, were inserted into the multi-cloning sites of pIZT/V5-His using pEASY®-Uni Seamless Cloning and Assembly Kits (TransGen Biotech, Beijing, China), respectively.

The plasmids, which were used to silence *S. frugiperda ATG5*, were constructed as described by Katsuma *et al*. [15]. Briefly, the fragments of *S. frugiperda ATG5* (dsRNA1-U and dsRNA1-D) were separately amplified from cDNA of Sf9 cells using the primer pair ATG5i1-Up-F/ATG5i1-Up-R and ATG5i1-Do-F/ATG5i1-Do-R, respectively. The dsRNA1-U and dsRNA1-D were inserted into *Kpn*I–*Age*I digested plasmid pIZT/V5-His using a pEASY®-Uni Seamless Cloning and Assembly Kit, creating the recombinant plasmid pIZT-ATG5i1. The primer pairs ATG5i2-Up-F/ATG5i2-Up-R and ATG5i2-Do-F/ATG5i2-Do-R were used to construct the recombinant plasmid pIZT-ATG5i2. Primers used in vector construction are listed in the (Table 1). The purification of Vip3Aa was performed as described previously [11]. The finally concentration of Vip3Aa used to treat Sf9 cells in this study was 40 μg/mL.

**Table 1.** Primers used in the present study

Primers	Primer Sequence
pIZT-A8-F	GCATGGACGAGCTGTACAAGATGAAATTCCAATATAAAGAAG
pIZT-A8-R	AATGGTGATGGTGATGATGATTAATAATATCCATAAACATTTTCATC
pIZT-GFP-F	TCGAATTTAAAGCTTGGTACATGGTGAGCAAGGGCGAGGAGCTG
pIZT-GFP-R	CTTCTTTATATTGGAATTTCATCTTGTACAGCTCGTCCATGC
pIZT-RFP-F	AACACGTCAAGAGCTCGCGGATGCAGGCCTCTGCAGTCGAC
pIZT-RFP-R	TCGCCCTTGCTCACCATGTCACTAGTCTTGTACAGCTCGTCC
pIZT-A5-F	TCGAATTTAAAGCTTGGTACATGGCTAATGATCGAGAGGTT
pIZT-A5-R	AATGGTGATGGTGATGATGATTAAAAGACACACATATGAAGGA
ATG5i1-Up-F	TCGAATTTAAAGCTTGGTACGAAATAATGGAAATACAACAAC
ATG5i1-Up-R	GTGGTTGGATTATAATGGGGACGTCTTCTGGGAACTTAGT
ATG5i1-Do-F	TAAGTTCCCAGAAGACGTCCCCATTATAATCCAACCACAT
ATG5i1-Do-R	AATGGTGATGGTGATGATGAGAAATAATGGAAATACAACAAC
ATG5i2-Up-F	TCGAATTTAAAGCTTGGTACGCCCCTCAAATGGCACTACC
ATG5i2-Up-R	TTACAAAATGACAAATTCGCTAACAAGGCGCTGACTGCAT
ATG5i2-Do-F	GCAGTCAGCGCCTTGTTAGCGAATTTGTCATTTTGTAAAC
ATG5i2-Do-R	AATGGTGATGGTGATGATGAGCCCCTCAAATGGCACTACC

### Observation of the ultrastructure of Sf9 cells

Alexander *et al*. has described the observation of autophagy by Transmission electron microscopy (TEM) [16]. Briefly, after treatment with Vip3Aa (40 μg/mL) for 12, 24, 36, and 48 h, the cells were fixed overnight in 2% paraformaldehyde and 3% glutaraldehyde. After that, the cells were exposed to 1% osmic acid at 25°C for 1–1.5 h. Then, the cell samples were evaporated in a series of ethanol solutions (30%, 50%, 70%, and 90%), soaked, and embedded in EPON812. The cells were then sectioned at 60 nm thickness using an ultramicrotome. Ultrathin sections were counterstained with lead citrate and uranyl acetate. Finally, the sections were observed using TEM (JEOL-1200EX; JEOL USA, Inc., Peabody, MA, USA).

### Total protein extraction and western blotting analysis

Total protein extraction was operated as described in our previous study [11]. Briefly, the Sf9 cells were lysed in 400 μL RIPA buffer with protease and phosphatase inhibitors, and the cell lysate was set on ice for 15–20 min and centrifuged at 4°C, 14,000 × *g* for 15 min. Finally, the supernatant containing the total protein extraction was used for western blotting analysis.

The proteins were separated by 7.5% or 12% or 15% sodium dodecyl sulfate polyacrylamide gel electrophoresis (SDS-PAGE) and transferred onto polyvinylidene fluoride (PVDF) membranes. After blocking with 5% bovine serum albumin (BSA) or skim milk, the membranes were incubated with primary antibodies at 4°C for 12–16 h. Primary antibodies were anti-AMPK (1:500), anti-p-AMPK (Thr172) (1:300), TSC2 (1:500), p-TSC2 (Ser1387) (1:300), Atg5 (1:1000), Beclin1 (1:500), p62 (1:1000), LC3-I/II (1:500), mTOR (1:500), p-mTOR (Ser2448) (1:300), ULK1 (#A8529), p-ULK1 (Ser555) (1:300), p-ULK1 (Ser757) (1:300), p70S6K (1:500), p-p70S6K (Thr389) (1:200), and anti-β-actin (1:500). Then, the membranes were incubated with secondary antibodies at 25°C for 1–2 h. Finally, Immobilon Western Chemiluminescent HRP Substrate (Millipore, Milan, Italy) was used to detect the results.

### Insect cell transfection

Insect cell transfection have been reported before [9]. Recombinant plasmids for RNA interference or overexpression were transfected into Sf9 cells via Cellfectin® II Reagent and PLUS™ Reagent. Briefly, Sf9 cells (5 × 10^5^ cells/mL) were seeded into 25 cm^2^ flasks for 48 h. The transfection mixture was consist of recombinant plasmid (2 μg), Cellfectin® II Reagent (8 μL), and PLUS™ Reagent (5 μL).

After washing three times with phosphate-buffered saline (PBS), the transfection mixture supplemented with 3 mL Grace’s insect medium was gently added into the 25 cm^2^ flask. After 12 h, the transfection mixture was replaced by Sf-900 II SFM medium supplemented with 6% heat-inactivated FBS. The cells were cultured for another 48 h. Thereafter, the selection medium containing 300–500 μg/mL *Zeocin* was used to culture the cells for 2–3 weeks at 28°C. Finally, we observed the cells under a fluorescent microscope and examined the expression level of *ATG5* by quantitative real-time reverse transcription PCR (qRT-PCR) analysis.

### Intracellular ATP content detection

ATPlite 1step Luminescence Assay (PerkinElmer, Boston, MA, USA) was used to analyze the intracellular ATP content. The steps for this analysis were as follows. The cell suspension (2.5 × 10^4^ cells) was added into a 96-well white plate and cultivated 12–16 h at 28°C. The cells were treated with Vip3Aa for 6, 12, 24, 36, 48, and 60 h, respectively. Dialysis buffer was used as a control. First, we produced the ATP test liquid by mixing ATPLite buffer and substrate powder. After washing the 96-well plate with PBS three times, the cell lysate and the ATP test liquid were added to the 96-well plate in turn. Then, the 96-well plate was placed on a horizontal shaker to mix the ATP reaction system. The luminosity intensity was tested by a multifunctional microplate reader (PerkinElmer, Boston, MA, USA).

### ATG5 expression level analysis

*ATG5* expression level was quantitative tested by qRT-PCR. The primers used in detecting the expression of *ATG5* are listed in (Table 2). cDNA was synthesized with the Primescript^TM^ RT reagent kit with gDNA Eraser (Takara, Dalian, China). Quantitative detection of *ATG5* expression level was determined with a SYBR® Premix Ex Taq™ (Takara) using primer pairs Sf-A5-RT-F/Sf-A5-RT-R in an Applied Biosystems 7900HT Real-Time PCR System (Applied Biosystems, Carlsbad, CA, USA). *GAPDH* was used as a standardized control by the 2^−*ΔΔCt*^ method.

**Table 2.** Primers used in the present study

Primers	Primer Sequence
Sf-RT-A5-F	GTTCCCAGAAGACGTCCTAC
Sf-RT-A5-R	GATTGTGGTCTTTCTTCTGC
Sf-GAPDH-F	GTGCCCAGCAGAACATCAT
Sf-GAPDH-R	GGAACACGGAAAGCCATAC

### Sf9 cell viability analysis

Sf9 cell viability was operated as described in our previous study [11]. Briefly, the cell suspension (2.5 × 10^4^ cells) was added into a 96-well plate and cultivated 12–16 h at 28°C. The old medium was replaced with a new medium containing Vip3Aa (40 μg/mL) and the cells were cultured for another 24 h or 48 h. Finally, the cell viability was tested according to the instructions of CCK-8 counting Kit (Dojindo, Kumamoto, Japan).

### Statistical analysis

The significance was tested by one-way analysis of variance using Student’s t test. If the p-value was ≤ 0.05, the results were considered significant.

## Results

### Vip3Aa induces autophagy in Sf9 cells

Jiang *et al*. and Hernández-Martinez *et al*. successfully demonstrated that Vip3Aa induced apoptosis in insect cells [9]. In view of the crosstalk between apoptosis and autophagy, we continued to investigate whether autophagy was actived during Vip3Aa treatment in Sf9 cells.

**Figure 1.**
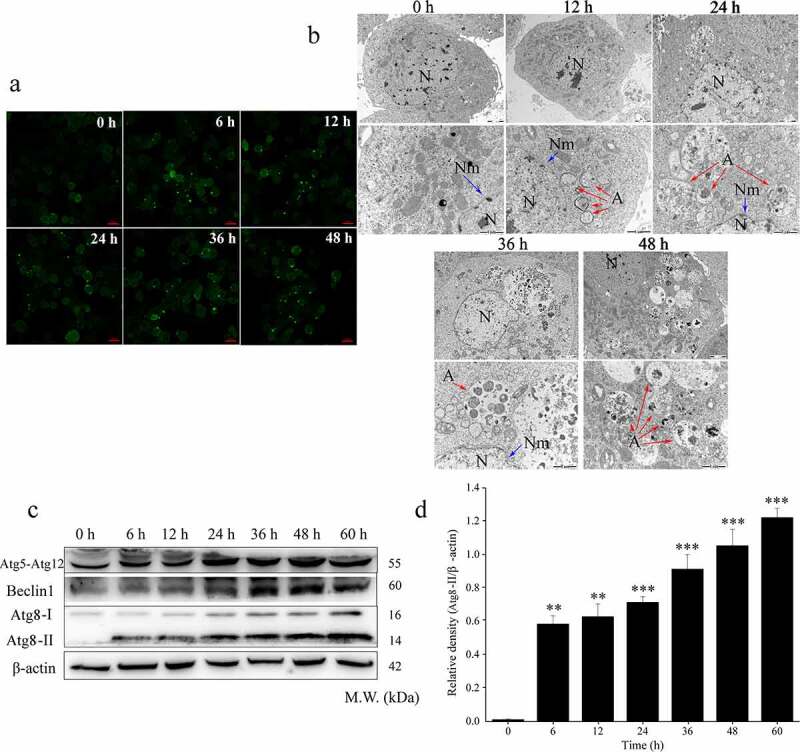
Vip3Aa induced autophagy in Sf9 cells. (a) Vip3Aa treatment induced the appearance Atg8 fluorescent spots in Sf9 cells. Scale bar, 20 μm. (b) Representative TEM photographs of autophagosome ultrastructures in Vip3Aa-treated Sf9 cells. N, nucleus. Nm, nuclear membrane (blue arrows). A, autophagosomes (red arrows). Scale bar, 1 μm. (c) The protein levels of autophagy-related proteins were detected using western blotting. (d) Relative densitometry analysis of Atg8-II/β-actin. Significant differences from the controls are shown as *p < 0.05, **p < 0.01, and ***p < 0.001

In the present study, we first explored whether Vip3Aa was able to induce autophagy in Sf9 cells using GFP-Atg8 punctation and TEM. The results (Figure 1a) showed that Vip3Aa caused Sf9 cells to accumulate GFP-Atg8 puncta at 6 h. GFP-Atg8 puncta were also detected in Vip3Aa-treated cells from 12 h to 48 h. As shown in (Figure 1b), the ultrastructural features of autophagy were obvious after Vip3Aa treatment. In addition, Vip3Aa increased the number of autophagic vesicles. Taken together, these findings confrmed that Vip3Aa induces autophagy in Sf9 cells.

### Vip3Aa induced autophagosome formation-related protein expression

Autophagy-related proteins (Atg) have been identified as the core machinery of autophagosome biogenesis. To explore the formation of autophagosomes during Vip3Aa treatment, we detected the protein levels of proteins related to autophagosoms formation, such as Atg5-Atg12, Beclin 1, and Atg8. In the process of autophagy, Atg8-I in the cytoplasm is converted into lipid-bound Atg8-II, which then accumulates on the membrane of autophagosomes. Therefore, the amount of Atg8-II correlates positivelyd with the number of autophagosomes [17]. As illustrated in (Figure 1c and d), Atg8-II was detected after Vip3Aa treatment for 6 h. With prolonged Vip3Aa treatment time, the level of Atg8-II increased to varying degrees. Morever, we observed that the Atg5-Atg12 and Beclin1 levels also increased with prolonged treatment time. These results proved that autophagosomes were formed during the entire process of Vip3Aa treatment, and the number of autophagosomes increased with the prolongation of treatment.

### Vip3Aa reduced ATP content in Sf9 cells

**Figure 2.**
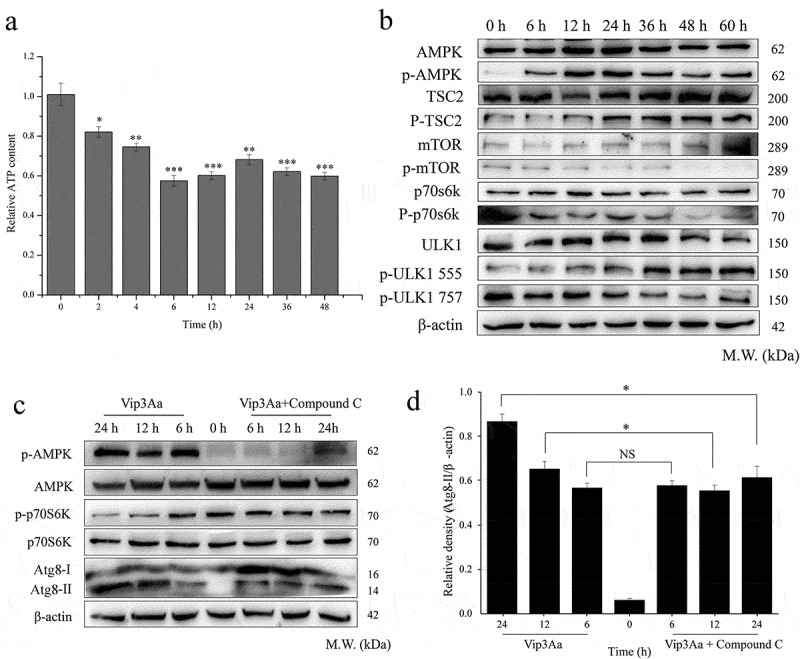
Vip3Aa induces autophagy through the AMPK-mTOR-ULK1 pathway in Sf9 cells. (a) Effect of Vip3Aa on the ATP content in Sf9 cells. (b) The levels of key signal molecules in the AMPK-mTOR-ULK1 pathway were detected by western blotting. (c) Effect of Compound C on the AMPK-mTOR-ULK1 pathway of Vip3A treated Sf9 cells. (d) Relative densitometry analysis of Atg8/β-actin. Significant differences from the controls are shown as *p < 0.05, **p < 0.01, and ***p < 0.001

To investigate whether energy metabolism is related to the autophagy response during Vip3Aa treatment, we detected the ATP content in Sf9 cells using an ATPlite 1step Luminescence Assay. As shown in (Figure 2a), compared with that in the control group (0 h), Vip3Aa treatment reduced the ATP levels in Sf9 cells by varying degrees. When treated with Vip3Aa for 6 h, the ATP level in Sf9 cells was the lowest (57.5% of the control group). When treated for 24 h, the ATP content was slightly higher than that at 6 h, at 68.1% of the control group. After Vip3Aa treatment for 24 h, the ATP level showed a downward trend again. These results confirmed that cellular ATP was indeed decreased in Vip3Aa-treated cells. Thus, Vip3Aa treatment could cause energy stress in Sf9 cells.

### Vip3Aa induces autophagy through the AMPK-MTOR-ULK1 pathway

Vip3Aa could induce the formation of autophagosomes; therefore, we continued to explore the autophagy signaling pathway. Vip3Aa reduced intracellular ATP levels, which might activate the protein kinase AMPK, a key regulator of autophagy, is modulated by energy metabolism [18]. Shang *et al*. revealed that AMPK can regulate mTOR negatively, which is conducive to the formation of autophagosomes [19]. We aimed to determine whether AMPK and mTOR are involved in Vip3Aa-induced autophagy. First, we detected the phosphorylation of AMPK and mTOR. As shown in (Figure 2b), Vip3Aa increased the phosphorylation of AMPK at Thr^172^ in Sf9 cells, while the phosphorylation of mTOR at Ser^2448^ decreased significantly.

ULK1 and p70S6K are direct targets of mTOR. mTOR can inhibit ULK1 activity by phosphorylation of ULK1 at Ser^757^, and activates p70S6K by phosphorylation of p70S6K at Thr^389^. The levels of p-ULK1^757^ and p-p70S6K also decreased with prolonged Vip3Aa treatment time (Figure 2b). These results indicated that Vip3Aa activated AMPK and inactivated mTOR.

Compound C, an AMPK specific inhibitor, was used to explore the effect of AMPK activation on mTOR activity and autophagosome formation. Compared with the Compound C untreated group, the activation of AMPK was significantly inhibited by Compound C, and this inhibition of AMPK also partially reduced the content of p-p70S6K and Atg8-II, especially when the cells were treated with Vip3Aa for 12 and 24 h (Figure 2c and d). Inhibition of AMPK partially reversed the inhibition of mTOR and reduced the content of Atg8-II. These results suggested that Vip3Aa could induce autophagy by activating the AMPK-mTOR-ULK1 pathway in Sf9 cells.

### Vip3Aa affects the degradation of autophagosomes in Sf9 cells

**Figure 3.**
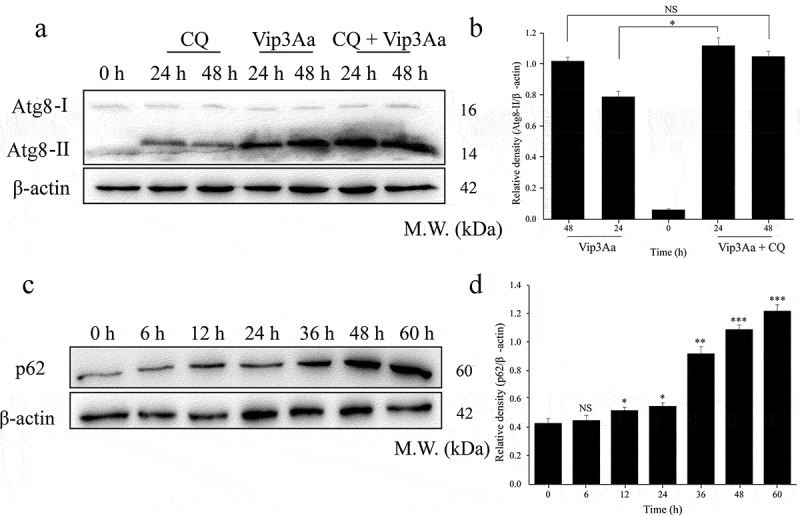
Vip3Aa can inhibit autophagic degradation in Sf9 cells. (a) The protein levels of Atg8-II/Atg8-I were detected by western blotting. (b) Relative densitometry analysis of Atg8-II/β-actin. (c) The protein levels of p62 were detected by western blotting. (d) Relative densitometry analysis of p62/β-actin. Cells were preincubated with CQ for 2 h. Significant differences from the controls are shown as *p < 0.05, **p < 0.01, and ***p < 0.001

In our previous work, we found that Vip3Aa increased the lysosomal pH in Sf9 cells [11]. Lysosomes are the degradation chambers of autophagosomes and are the key to autophagy circulation. Therefore, we speculated that Vip3Aa might affect the degradation of autophagosomes. Consequently, we used the lysosomal inhibitor chloroquine (CQ) to inhibit the fusion of autophagosomes and lysosomes, and detected the changes in Atg8-II protein levels. This Atg8-II turnover analysis method could estimate the amount of autophagosomes degraded by lysosomes [20]. As shown in (Figure 3a and b), compared with Vip3Aa treatment alone for 24 h, the content of Atg8-II increased significantly when the cells were treated with Vip3Aa and CQ, and the difference between them represented the lysosomal degraded autophagosomes. However, the amount of Atg8-II did not increase significantly when the treatment time prolonged to 48 h. These results showed Vip3Aa treatment might lead to impaired autophagosome clearance.

p62 is an autophagy specific substrate, which can be degraded during autophagy circulation. Our results (Figures 3c and d) showed the protein level of p62 increased with the prolongation of Vip3Aa treatment time. These results further proved that Vip3Aa affected the degradation of autophagosomes in Sf9 cells. Namely, Vip3Aa impeded autophagy circulation.

**Figure 4.**
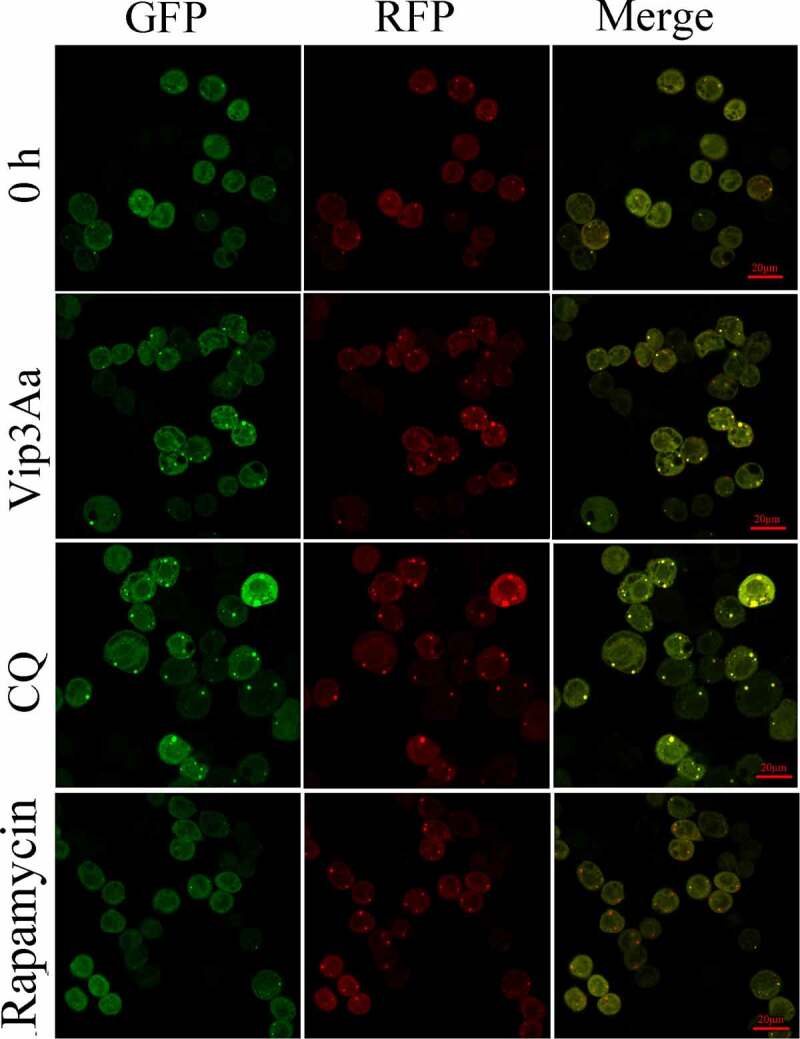
Atg8 fluorescence results in Sf-RGatg8 cells. Sf-RGatg8 cells were treated with CQ and Rapamycin for 24 h in. Scale bar, 20 μm

To investigate autophagy flux in Sf9 cells treated with Vip3Aa for 24 h, we constructed cell line Sf-RGatg8, which expressed RFP-GFP-Atg8. In Sf-RGatg8 cells, red fluorescent protein (RFP) and green fluorescent protein (GFP) are connected in series. In the normal autophagy process, the acidic environment in the lysosome will cause quenching of GFP, and only red fluorescence will be detected. Conversely, if the autophagosome is not degraded in the lysosome, both RFP and GFP will remain, resulting in yellow fluorescence. As illustrated in (Figure 4), in rapamycin-treated Sf-RGatg8 cells, red fluorescence puncta increased, indicating autophagy activation and autophagosome degradation. CQ treatment resulted in an increase of yellow fluorescent spots, indicating that autophagosome degradation was inhibited. The results for the Vip3Aa-treated group were similar to those of the CQ-treated group, except that a small number of red fluorescent spots were also seen in Vip3Aa-treated cells. These results indicated that autophagy degradation had begun to be inhibited in Vip3Aa-treated Sf9 cells after 24 h.

### Autophagy protects Sf9 cells exposed to Vip3Aa

**Figure 5.**
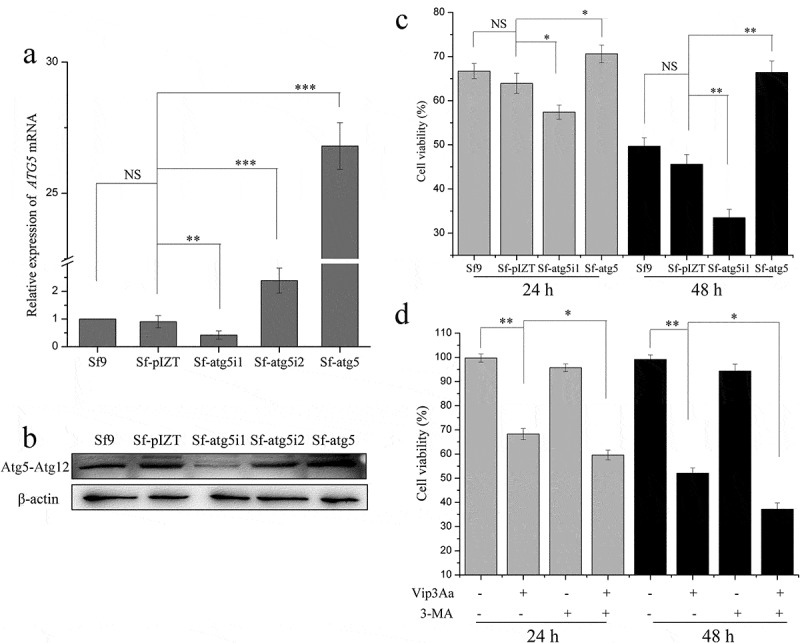
Autophagosome accumulation induced by Vip3Aa plays a pro-survival role in Sf9 cells. (a) The mRNA expression levels of *ATG5*. (b) The protein level of Atg5-Atg12, as detected by western blotting. (c) Effect of Atg5 knockdown or overexpression on cell viability. (d) Effect of 3-MA on cell viability. Significant differences from the controls are indicated as *p < 0.05, **p < 0.01, and ***p < 0.001

To study the role of autophagy in the toxic effects of Vip3Aa, we constructed an insect cell line that was knocked down or overexpressed Atg5, a pivotal protein for autophagosome forming. The knockdown and overexpression cell lines were named as Sf-atg5i1 and Sf-atg5, respectively (Figure 5a and b). As seen in (Figure 5c), when the cells was treated with Vip3Aa for 24 h, the survival rates of Sf9, Sf-atg5i1, and Sf-atg5 cells were 66.7%, 57.4%, and 70.6%, respectively. When the action time was extended to 48 h, the survival rates of Sf9, Sf-atg5i1, and Sf-atg5 cells were 49.7%, 33.5%, and 66.4%, respectively. The cytotoxicity test indicated that when Atg5 was overexpressed, cell viability increased; and when Atg5 was knocked down, cell viability decreased. Moreover, the autophagy inhibitor 3-MA also increased the sensitivity of Sf9 cells to Vip3Aa (Figure 5d). These results indicated that autophagy plays a protective role in Sf9 cell death induced by Vip3Aa.

## Discussion

Vip3Aa can control Lepidoptera pests such as *S. frugiperda* and *Agrotis ypsilon* effectively. It is believed widely that Vip3Aa used alone, or in combination with other insecticidal proteins, can induce effective biological control. Therefore, to maximize the insecticidal potential of Vip3Aa, it is necessary to study its induced cell death mechanism. Previously, we showed that Vip3Aa exerts its cytotoxic effects on Sf9 cells through the activation of caspase-9/-3, which causes mitochondrial dysfunction [9,11]. Recently, we further revealed that the instability of the lysosome membrane caused by Vip3Aa contributes to activating caspase cascades [11]. In the present study, we observed that Vip3Aa could induce autophagy in Sf9 cells, and that autophagy might be related to the development of insect cell resistance.

The main function of autophagy in most cell types is considered to be an adaptive response to starvation, and it can degrade proteins and organelles damaged by oxidative stress, maintaining cell homeostasis [12]. Thus, autophagy is essential for cell survival. This study proved that Vip3Aa can induce the formation of autophagosomes. It was confirmed by observing cell ultrastructure, GFP-Atg8 puncta and detecting changes in autophagy-related protein content.

In a previous study, we found that Vip3Aa could enter Sf9 cells through macropinocytosis-related endocytosis, which is an energy-consuming process [21]. Moreover, we confirmed that Vip3Aa could induce mitochondrial dysfunction. We speculated that Vip3Aa might affect energy metabolism. As expected, we found that Vip3Aa caused a decrease in intracellular ATP levels and activated AMPK. mTOR is one of the key inhibitors of autophagy, which is positively regulated by upstream signals (such as growth factors and amino acids) and negatively regulated by AMPK [22]. Combined with the use of Compound C, a specific AMPK inhibitor, we showed that AMPK activation could inhibit the activity of mTOR in Vip3Aa-treated Sf9 cells. In other words, Vip3Aa could activate autophagy through the AMPK-mTOR pathway.

Autophagy begins in the ULK1/Atg1 complex and is evolutionarily conserved from yeast to mammals [23]. In mammals, mTOR inhibits ULK1 by phosphorylation of ULK1 at Ser^757^; however, AMPK can activate ULK1 by phosphorylation of ULK1 at Ser^555^ [24]. In Sf9 cells, we found that Vip3Aa caused increased levels of ULK1 phosphorylated at Ser^555^ in a time-dependent manner; but the level of ULK1 phosphorylated at Ser^757^ decreased. This result also supported the view that Vip3Aa could activate AMPK and inactivate mTOR.

Autophagy is known to promote cell death or cell survival depending on the nature of the stimulus and the cellular environment [25]. To investigate the effect of Vip3Aa on autophagy, Atg8-II turnover analysis was used to detect autophagy flux. The results confirmed that autophagosome degradation was inhibited after Vip3Aa treatment for 48 h. We also found that the increase in Atg8-II caused by Vip3Aa was accompanied by increased of p62 levels after 12 h of treatment, which indicated that Vip3Aa might have begun to inhibit autophagy flux. The results of RFP-GFP-Atg8 fluorescent spot detection confirmed that autophagic flux was indeed blocked by Vip3Aa. Moreover, studies have shown that blocking the autophagosome degradation step might lead to the accumulation of enlarged and unstable autophagic vesicles [26]. In the present study, TEM observations revealed that large-diameter (approximately 2 μm) autophagic vesicles appeared in Sf9 cells after Vip3Aa treatment for 24 h (Figure 1b). This also confirmed that Vip3Aa impaired autophagy flux, leading to autophagosome accumulation. Our previous study found that when Sf9 cells were treated with Vip3Aa for 12 h, the stability of the lysosome began to be destroyed. Damaged lysosomes would lead to autophagosome accumulation. Autophagy signals were detected at the early stage of Vip3Aa treatment (6 h), and we hypothesized that autophagy represented the response of Sf9 cells to Vip3Aa stress. However, Vip3Aa could inhibit this response by attacking lysosomes.

To explore the relationship between autophagy and Sf9 cell death, we knocked down and overexpressed *ATG5* in Sf9 cells. We found that decreased *ATG5* expression increased the sensitivity of Sf9 cells to Vip3Aa, while overexpression of *ATG5* reduced the mortality of Vip3Aa-treated cells. These results indicated that autophagy plays a protective role in Vip3Aa-treated cells. The effect of 3-MA on Vip3Aa-treated Sf9 cells also confirmed the role of autophagy in protecting Sf9 cells.

Autophagy attenuates the enhancement of death signals by degrading damaged organelles, such as damaged mitochondria and lysosomes, which are closely associated with cell death. Yu et al. showed that autophagy could be induced to limit the cell damage caused by the disrupted organelles [27]. Our previous study found that Vip3Aa caused damage to lysosomes and mitochondria. Furthermore, in the present study, damaged mitochondria and lysosomes could be seen in the autophagosomes (Figure 1b). Thus, we speculated that autophagy could degrade damaged organelles, delay the occurrence of cell death, and played an antagonistic role against Vip3Aa toxicity in the initial stage of Vip3Aa treatment (12 h). However, with extended treatment time, the damage caused by Vip3Aa to lysosomes would be aggravated. In turn, the damaged mitochondria would also act on the lysosome, attacking the membrane stability of the lysosome, and resulting in more damaged lysosomes. The occurrence of these events would further inhibit autophagy circulation. Therefore, this autophagic salvage response in Sf9 cells might only operate under certain conditions.

Lysosomes are the only sites at which autophagosomes are degraded [28]. Our results suggested that Vip3Aa attacks lysosomes and hinder autophagosome degradation [11]. As the Vip3Aa concentration increased, the number of damaged lysosomes increased. In addition, Sf9 cells will continue to activate the autophagic salvage response under conditions of Vip3Aa pressure. These events will cause further autophagosome accumulation, which might turn autophagy into a destructive process [29,30]. Moreover, increased autophagy signaling will also increase the burden of lysosomes, which would lead to lysosomal membrane permeabilization [31]. Thus, the destruction in the later stage of autophagy leads to the excessive accumulation of autophagic vacuoles. In this case, too many autophagosomes might lead to cell death.

There are few reports on the mechanism of Vip3Aa resistance. Crickmore *et al*. proposed that the loss of receptor function caused by the intracellular response might be a potential mechanism of insecticidal toxin resistance [32]. Some studies tried to the explore lepidopteran gut transcriptome to determine the resistance mechanism of Vip3Aa [33,34]. However, no conclusive results were obtained for the resistance mechanism of Vip3Aa. In the present study, we found that autophagy in Sf9 cells might be a rescue response to Vip3Aa stress and could antagonize the virulence of Vip3Aa. We speculated that this autophagic salvage response might play a role in insect resistance development.

Autophagy antagonizes the Sf9 cell death induced by Vip3Aa; however, the relationship between autophagy and Vip3Aa-induced Sf9 cell apoptosis remains unclear. In addition, when the concentration of Vip3Aa increased, lysosomal damage might be aggravated, and the autophagosome accumulation induced by Vip3Aa might not be conducive to cell survival. Therefore, to explore the mechanism of Vip3Aa’s action in Sf9 cells more deeply, further research is required.

In conclusion, we found that Vip3Aa initiated autophagy through the AMPK-MTOR-ULK1 pathway in Sf9 cells. We also found that autophagy had an antagonistic effect on the Sf9 cytotoxicity induced by Vip3Aa. However, with extended treatment time, Vip3Aa could affect the degradation of autophagosomes, leading to impaired autophagy flux. Our findings provide a better understanding of Vip3Aa’s insecticidal mechanism, promoting future exploitation of its full insecticidal potential and techniques delay its induced resistance.

## Supplementary Material

Supplemental Material

## References

[1] James C. Global status of commercialized biotech/GM crops: 2017. Brief No 53 ISAAA. Ithaca, NY, USA; 2018.

[2] Edgerton MD, Fridgen J, Anderson JR, et al. Transgenic insect resistance traits increase corn yield and yield stability. Nat Biotechnol. 2012;30:493–496. 22678382 10.1038/nbt.2259

[3] Hutchison WD, Burkness EC, Mitchell PD, et al. Areawide suppression of European corn borer with Bt maize reaps savings to non-Bt maize growers. Science. 2010;330:222–225. 20929774 10.1126/science.1190242

[4] Lu Y, Wu K, Jiang Y, et al. Widespread adoption of Bt cotton and insecticide decrease promotes biocontrol services. Nature. 2012;487:362–365. 22722864 10.1038/nature11153

[5] Lee MK, Walters FS, Hart H, et al. The mode of action of the *Bacillus thuringiensis* vegetative insecticidal protein Vip3A differs from that of Cry1Ab delta-endotoxin. Appl Environ Microbiol. 2003;69:4648–4657. 12902253 10.1128/AEM.69.8.4648-4657.2003 PMC169065

[6] Sena JA, Hernández-Rodríguez CS, Ferré J. Interaction of *Bacillus thuringiensis* Cry1 and Vip3A proteins with *Spodoptera frugiperda* midgut binding sites. Appl Environ Microbiol. 2009;75:2236–2237. 19181834 10.1128/AEM.02342-08 PMC2663230

[7] Han S, Craig JA, Putnam CD, et al. Evolution and mechanism from structures of an ADP-ribosylating toxin and NAD complex. Nat Struct Biol. 1999;6:932–936. 10504727 10.1038/13300

[8] Kunthic T, Watanabe H, Kawano R, et al. pH regulates pore formation of a protease activated Vip3Aa from *Bacillus thuringiensis*. Biochimica Et Biophysica Acta Biomembranes. 1999;6(11):2234–2241. 10.1016/j.bbamem.2017.08.018 28865796

[9] Jiang K, Mei SQ, Wang TT, et al. Vip3Aa induces apoptosis in cultured *Spodoptera frugiperda* (Sf9) cells. Toxicon. 2016;120:49–56. 27476462 10.1016/j.toxicon.2016.07.019

[10] Hernández-Martínez P, Gomis-Cebolla J, Ferré J, et al. Changes in gene expression and apoptotic response in *Spodoptera exigua* larvae exposed to sublethal concentrations of Vip3 insecticidal proteins. Sci Rep. 2017;7:16245. 29176692 10.1038/s41598-017-16406-1 PMC5701239

[11] Hou XY, Han L, An BJ, et al. Mitochondria and Lysosomes Participate in Vip3Aa-Induced *Spodoptera frugiperda* Sf9 Cell Apoptosis. Toxins (Basel). 2020;12. 10.3390/toxins12020116 PMC7076775 32069858

[12] Mukhopadhyay S, Panda PK, Sinha N, et al. Autophagy and apoptosis: where do they meet? Apoptosis. 2014;19:555–566. 24415198 10.1007/s10495-014-0967-2

[13] Scherz-Shouval R, Shvets E, Fass E, et al. Reactive oxygen species are essential for autophagy and specifically regulate the activity of Atg4. Embo J. 2007;26:1749–1760. 17347651 10.1038/sj.emboj.7601623 PMC1847657

[14] Dodson M, Darley-Usmar V, Zhang J. Cellular metabolic and autophagic pathways: traffic control by redox signaling. Free Radical Bio Med. 2013;63:207–221. 23702245 10.1016/j.freeradbiomed.2013.05.014 PMC3729625

[15] Katsuma S, Daimon T, Mita K, et al. Lepidopteran ortholog of Drosophila breathless is a receptor for the baculovirus fibroblast growth factor. J Virol. 2006;80:5474–5481. 16699027 10.1128/JVI.00248-06 PMC1472154

[16] Alexander DE, Ward SL, Mizushima N, et al. Analysis of the role of autophagy in replication of herpes simplex virus in cell culture. J Virol. 2007;81:12128–12134. 17855538 10.1128/JVI.01356-07 PMC2169004

[17] Mizushima N, Yoshimori T. How to interpret LC3 immunoblotting. Autophagy. 2007;3:542–545. 17611390 10.4161/auto.4600

[18] Meley D, Bauvy C, Houben-Weerts JH, et al. AMP-activated protein kinase and the regulation of autophagic proteolysis. J Biol Chem. 2006;281:34870–34879. 16990266 10.1074/jbc.M605488200

[19] Shang L, AMP WX. K and mTOR coordinate the regulation of Ulk1 and mammalian autophagy initiation. Autophagy. 2011;7:924–926. 21521945 10.4161/auto.7.8.15860

[20] Yuan H, Perry CN, Huang C, et al. LPS-induced autophagy is mediated by oxidative signaling in cardiomyocytes and is associated with cytoprotection. Am J Physiol-Heart C. 2009;296:H470–479. 10.1152/ajpheart.01051.2008 PMC2643899 19098111

[21] Jiang K, Hou XY, Tan TT, et al. Scavenger receptor-C acts as a receptor for *Bacillus thuringiensis* vegetative insecticidal protein Vip3Aa and mediates the internalization of Vip3Aa via endocytosis. PLoS Path. 2018;14:e1007347. 10.1371/journal.ppat.1007347 PMC6191154 30286203

[22] Laplante M, Sabatini DM. mTOR signaling in growth control and disease. Cell. 2012;149:274–293. 22500797 10.1016/j.cell.2012.03.017 PMC3331679

[23] Beau I, Mehrpour M, Codogno P. Autophagosomes and human diseases. Int J Biochem Cell B. 2011;43:460–464. 10.1016/j.biocel.2011.01.006 21256243

[24] Alers S, Löffler AS, Wesselborg S, et al. Role of AMPK-mTOR-Ulk1/2 in the regulation of autophagy: cross talk, shortcuts, and feedbacks. Mol Cell Biol. 2012;32:2–11. 22025673 10.1128/MCB.06159-11 PMC3255710

[25] Anding AL, Baehrecke EH. Autophagy in Cell Life and Cell Death. Curr Top Dev Biol. 2015;114:67–91. 26431564 10.1016/bs.ctdb.2015.07.012

[26] Gonzalez P, Mader I, Tchoghandjian A, et al. Impairment of lysosomal integrity by B10, a glycosylated derivative of betulinic acid, leads to lysosomal cell death and converts autophagy into a detrimental process. Cell Death Differ. 2012;19:1337–1346. 22343715 10.1038/cdd.2012.10 PMC3392623

[27] Yu L, McPhee CK, Zheng L, et al. Termination of autophagy and reformation of lysosomes regulated by mTOR. Nature. 2010;465:942–946. 20526321 10.1038/nature09076 PMC2920749

[28] Saftig P, Klumperman J. Lysosome biogenesis and lysosomal membrane proteins: trafficking meets function. Nat Rev Mol Cell Bio. 2009;10:623–635. 19672277 10.1038/nrm2745

[29] Morgan MJ, Gamez G, Menke C, et al. Regulation of autophagy and chloroquine sensitivity by oncogenic RAS in vitro is context-dependent. Autophagy. 2014;10:1814–1826. 25136801 10.4161/auto.32135 PMC4198365

[30] Shen J, Zheng H, Ruan J, et al. Autophagy inhibition induces enhanced proapoptotic effects of ZD6474 in glioblastoma. Br J Cancer. 2013;109:164–171. 23799852 10.1038/bjc.2013.306 PMC3708568

[31] Yao XF, Cao J, Xu LM, et al. Perfluorooctane sulfonate blocked autophagy flux and induced lysosome membrane permeabilization in HepG2 cells. Food Chem Toxicol. 2014;67:96–104. 24561269 10.1016/j.fct.2014.02.017

[32] Crickmore N. *Bacillus thuringiensis* resistance in Plutella - too many trees? Curr Opin Insect Sci. 2016;15:84–88. 27436736 10.1016/j.cois.2016.04.007

[33] Bel Y, Jakubowska AK, Costa J, et al. Comprehensive analysis of gene expression profiles of the beet armyworm *Spodoptera exigua* larvae challenged with *Bacillus thuringiensis* Vip3Aa toxin. PloS One. 2013;8:e81927. 24312604 10.1371/journal.pone.0081927 PMC3846680

[34] Song F, Chen C, Wu S, et al. Transcriptional profiling analysis of *Spodoptera litura* larvae challenged with Vip3Aa toxin and possible involvement of trypsin in the toxin activation. Sci Rep. 2016;6:23861. 27025647 10.1038/srep23861 PMC4812304

