# Supplementary material for: Autophagy induced by Vip3Aa has a pro-survival role in Spodoptera frugiperda Sf9 cells
Source: Virulence. 2021 Jan 28;12(1):509–19. doi: 10.1080/21505594.2021.1878747 (PMC7849784; doi:10.1080/21505594.2021.1878747)
Supplement: Supplemental Material [file KVIR_A_1878747_SM0871.pdf]

Supplementary Information

1. Amino acid sequence alignment of AMPK protein between *Homo Sapiens* and *Spodoptera frugiperda* (Query: *Homo Sapiens*; Sbjct: *Spodoptera frugiperda*).

5'-AMP-activated protein kinase catalytic subunit alpha-2-like isoform X4 [Spodoptera frugiperda]

Sequence ID: XP_035447912.1 Length: 512 Number of Matches: 1

Range 1: 15 to 512 GenPept Graphics ▼ Next Match ▲ Previous Match

Score	Expect	Method	Identities	Positives	Gaps
699 bits(1804)	0.0	Compositional matrix adjust.	353/548(64%)	413/548(75%)	56/548(10%)

```
Query  11   VKIGHYVLGDTLGVGTFGKVKIGEHQLTGHKVAVKILNRQKIRSLDVVGKIKREIQNLKL  70
            VKIGHY LG TLGVGTFGKVKIGEHQLT HKVAVKILNRQKI+SLDVVGKI+REIQNLKL
Sbjct  15   VKIGHYTLGATLGVGTFGKVKIGEHQLTKHKVAVKILNRQKIKSLDVVGKIRREIQNLKL  74

Query  71   FRHPHIIKLYQVISTPTDFFMVMEYVSGGELFDYICKHGRVEEMEARRLFQQILSAVDYC  130
            FRHPHIIKLYQVISTPTD FM+MEYVSGGELFDYI K G+++E EARR FQQI+S VDYC
Sbjct  75   FRHPHIIKLYQVISTPTDIFMIMEYVSGGELFDYIVKRGKLQEHEARRFFQQIISGVDYC  134

Query  131  HRHMVVHRDLKPENVLLDAHMNAKIADFGLSNMMSDGEFLRTSCGSPNYAAPEVISGRLY  190
            HRHM+VHRDLKPEN+LLD +M+ KIADFGLSNMM DGEFLRTSCGSPNYAAPEVISG+LY
Sbjct  135  HRHMIVHRDLKPENLLLDHNMHVKIADFGLSNMMMDGEFLRTSCGSPNYAAPEVISGKLY  194

Query  191  AGPEVDIWSCGVILYALLCGTLPFDDEHVPTLFKKIRGGVFYIPEYLNRSVATLLMHMLQ  250
            AGPEVD+WSCGVILYALLCGTLPFDDEHVPTLF+KI+ G+F IPEYLN+SV +LL  MLQ
Sbjct  195  AGPEVDVWSCGVILYALLCGTLPFDDEHVPTLFRKIKSGIFPIPEYLNKSVVSLLCMMLQ  254

Query  251  VDPLKRATIKDIREHEWFKQDLPSYLFPEDPSYDANVIDDEAVKEVCEKFECTESEVMNS  310
            VDP+KRA+I+D+++HEWF++DLP YLFP     D++VID EA+ EVC+KF   E EV N+
Sbjct  255  VDPMKRASIEDVKKHEWFQKDLPGYLFPSPVEQDSSVIDTEAISEVCDKFGVKEHEVHNA  314

Query  311  LYSGDPQDQLAVAYHLIIDNRRIMNQAS-----EFYLAS-SPPSGSFMDDSAMHIPPGLK  364
            L SGDP DQLA+AYHLIIDN+RI ++A+     +FY+AS SPP+ S   ++A       +
Sbjct  315  LLSGDPHDQLAIAYHLIIDNKRIADEAAKAEIKDFYVASNSPPAAS---ETA-------R  364

Query  365  PHPERMPPLIADSPKARCPLDALNTTKPKSLAVKKAKWHLGIRSQSKPYDIMAEVYRAMK  424
            PHPER+ P    P  +         K +   VK+AKWHLGIRSQSKP DIM EV+RAMK
Sbjct  365  PHPERIAP----HPHPQ-------QDKARGTPVKRAKWHLGIRSQSKPNDIMLEVFRAMK  413

Query  425  QLDFEWKVVNAYHLRVRRKNPVTGNYVKMSLQLYLVDNRSYLLDFKSIDDEVVEQRSGSS  484
             LD+EWKV+N YH+RVR  N +T  YVKMSLQLY VD +SYLLDFKS+  E
Sbjct  414  ALDYEWKVINPYHVRVRTLNKMTEKYVKMSLQLYQVDYKSYLLDFKSLSGE---------  464

Query  485  TPQRSCSAAGLHRPRSSFDSTTAESHSLSGSLTGSLTGSTLSSVSPRLGSHTMDFFEMCA  544
                        +  S  ++ +    +       S TG          G HTM+FFEMCA
Sbjct  465  ------------KEDSDEEAASPLVAAAPPPPPASPTGPQ--------GHHTMEFFEMCA  504

Query  545  SLITTLAR  552
            +LI  LAR
Sbjct  505  ALIIQLAR  512
```

Note: The red boxes in the figure above are the predicted antigen epitopes, and the predicted URL is http://imed.med.ucm.es/Tools/antigenic.pl.

2. Amino acid sequence alignment of TSC2 protein between *Homo Sapiens* and *Spodoptera frugiperda* (Query: *Homo Sapiens*; Sbjct: *Spodoptera frugiperda*).

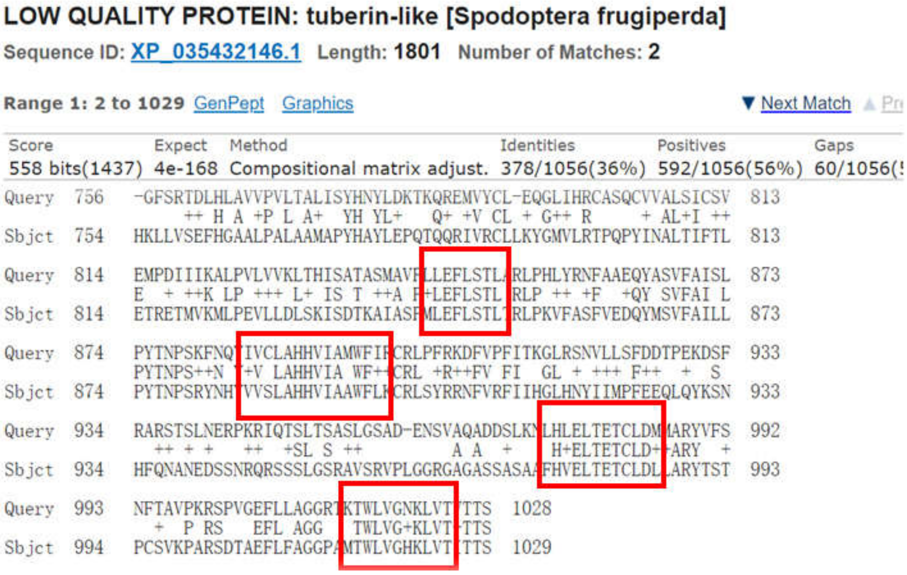

LOW QUALITY PROTEIN: tuberin-like [Spodoptera frugiperda]

Sequence ID: XP_035432146.1 Length: 1801 Number of Matches: 2

Range 1: 2 to 1029 GenPept Graphics ▼ Next Match ▲ Pr

Score	Expect	Method	Identities	Positives	Gaps
558 bits(1437)	4e-168	Compositional matrix adjust.	378/1056(36%)	592/1056(56%)	60/1056(

```
Query  756   -GFSRTDLHLAVVPVLTALISYHNYLDKTKQREMVYCL-EQGLIHRCASQCVVALSICSV  813
                  ++ H A +P L A+  YH YL+   Q+ +V CL + G++ R      + AL+I ++
Sbjct  754   HKLLVSEFHGAALPALAAMAPYHAYLEPQTQQRIVRCLLKYGMVLRTPQPYINALTIFTL  813

Query  814   EMPDIIIKALPVLVVKLTHISATASMAVFLLEFLSTLARLPHLYRNFAAEQYASVFAISL  873
             E  + ++K LP +++ L+ IS T ++A F+LEFLSTL RLP ++ +F  +QY SVFAI L
Sbjct  814   ETRETMVKMLPEVLLDLSKISDTKAIASFMLEFLSTLTRLPKVFASFVEDQYMSVFAILL  873

Query  874   PYTNPSKFNQTIVCLAHHVIAMWFIRCRLPFRKDFVPFITKGLRSNVLLSFDDTPEKDSF  933
             PYTNPS++N T+V LAHHVIA WF++CRL +R++FV FI  GL + +++ F++  +  S
Sbjct  874   PYTNPSRYNHTVVSLAHHVIAAWFLKCRLSYRRNFVRFIIHGLHNYIIMPFEEQLQYKSN  933

Query  934   RARSTSLNERPKRIQTSLTSASLGSAD-ENSVAQADDSLKNLHLELTETCLDMMARYVFS  992
               ++ + +   ++  +SL S ++             A A  +    H+ELTETCLD++ARY  +
Sbjct  934   HFQNANEDSSNRQRSSSLGSRAVSRVPLGGRGAGASSASAAFHVELTETCLDLLARYTST  993

Query  993   NFTAVPKRSPVGEFLLAGGRTKTWLVGNKLVT[illegible]TTS  1028
               +  P RS   EFL AGG   TWLVG+KLVT+TTS
Sbjct  994   PCSVKPARSDTAEFLFAGGPAMTWLVGHKLVT[illegible]TTS  1029
```

Note: The red boxes in the figure above are the predicted antigen epitopes, and the predicted URL is http://imed.med.ucm.es/Tools/antigenic.pl.

3. Amino acid sequence alignment of Atg5 protein between *Homo Sapiens* and *Spodoptera frugiperda* (Query: *Homo Sapiens*; Sbjct: *Spodoptera frugiperda*).

autophagy protein 5-like [Spodoptera frugiperda]

Sequence ID: XP_035429947.1 **Length:** 264 **Number of Matches:** 1

Range 1: 1 to 264 GenPept Graphics ▼ Next Match ▲ Previous Match

Score	Expect	Method	Identities	Positives	Gaps
294 bits(753)	2e-100	Compositional matrix adjust.	141/273(52%)	193/273(70%)	12/273(4%)

```
Query  1    MTDDKDVLRDVWFGRIPTCFTLYQDEITE-REAEPYYLLLPRVSYLTLVTDKVKKHFQKV  59
            M  D++VLR+ W G++P CF L Q+EI E ++ + +Y+++PR+SY  LVTDK+K+HF +
Sbjct  1    MANDREVLREIWDGKLPICFQLAQEEIMEIQQPDAFYVMVPRLSYFPLVTDKMKRHFLRY  60

Query  60   MRQEDI-SEIWFEYEGTPLKWHYPIGLLFDLLASSS-ALPWNITVHFKSFPEKDLLHCPS  117
            + QE+  SE+W +Y G PLKWHYPIG L+DL    +   LPW++TVHF  FPE  LLHC +
Sbjct  61   ISQENSDSEMWLDYNGQPLKWHYPIGFLYDLYCGNDPQLPWHLTVHFTKFPEDVLLHCTN  120

Query  118  KDAIEAHFMSCMKEADALKHKSQVINEMQKKDHKQLWMGLQNDRFDQFWAINRKLMEYPA  177
            KD +EAHFMS +KEAD LKH+ QV++ MQKKDH QLW+GLQND+FDQFWAINR+LME
Sbjct  121  KDVVEAHFMSTVKEADVLKHRGQVMSTMQKKDHNQLWLGLQNDKFDQFWAINRRLMESHG  180

Query  178  EENGFRYIPFRIYQTTTERPFIQKLFRPVAADGQLHTLGDLLKEVCPSAIDPEDGEKKNQ  237
            +  GF++IP ++Y  + +    Q+L  P   DG   TL  ++ E+ P  +D        Q
Sbjct  181  DSEGFKHIPIKLY--SDDGTCSQRLVSPKNNDGSRKTLQQMIAELYPDKLDV-------Q  231

Query  238  VMIHGIEPMLETPLQWLSEHLSYPDNFLHISII  270
            +  HGI     +TPLQWLSEHLSYPDNFLH+ +
Sbjct  232  LRTHGIVIPTDTPLQWLSEHLSYPDNFLHMCVF  264
```

Note: The red boxes in the figure above are the predicted antigen epitopes, and the predicted URL is http://imed.med.ucm.es/Tools/antigenic.pl.

4. Amino acid sequence alignment of Beclin1 protein between *Homo Sapiens* and *Spodoptera frugiperda* (Query: *Homo Sapiens*; Sbjct: *Spodoptera frugiperda*).

beclin-1-like protein [Spodoptera frugiperda]

Sequence ID: XP_035451380.1 **Length:** 427 **Number of Matches:** 1

Range 1: 8 to 419 GenPept Graphics ▼ Next Match ▲ Previous Match

Score	Expect	Method	Identities	Positives	Gaps
433 bits(1114)	6e-150	Compositional matrix adjust.	233/436(53%)	292/436(66%)	26/436(5%)

```
Query  14   VSFVCQRCSQPLKLDTSFKILDRVTIQELTAPLLTTAQAKPGETQEEETNSGEEPFIETP  73
            V+F CQRC QPLKLD S   L   TI +L   +   +                ++  
Sbjct  8    VNFSCQRCLQPLKLDESLNNLGEHTIADLALQIRRNNE-----------------VDLD  49

Query  74   RQDGVSRRFIPPARMM-STESANSFTLIGEASDGGTMENLSRRLKVTGDLFDIMSGGTDV  132
             Q       ++PP RM  S   AN F +I   SDG     +L  +L V   LFD++S +DV
Sbjct  50   IQSTSLEHYVPPFRMSESGNGANGFMVI---SDGWETTSLGHQLHVKATLFDLLSNNSDV  106

Query  133  DHPLCEECTDTLLDQLDTQLNVTENECQNYKRCLEILEQMNED-DSEQLQMELKELALEE  191
            DHPLC+ECTDTLL+ +D QL  TE E ++Y    L+ LE    ED + E L+ EL +    E+
Sbjct  107  DHPLCDECTDTLLELMDNQLRQTEAEWKDYNDYLKKLEDDKEDLNLEGLEKELGDWKQEQ  166

Query  192  ERLIQELEDVEKNRKIVAENLEKVQAEAERLDQEEAQYQREYSEFKRQQLELDDELKSVE  251
             RL+QEL   ++K  K + E ++   + E ERL++E+  Y REY+ +++  + ++D++K  E
Sbjct  167  SRLLQELSALQKEEKAMKEEIDIQEREKERLEKEQDVYWREYTRYRKDLMTIEDQMKFYE  226

Query  252  NQMRYAQTQLDKLKKTNVFNATFHIWHSGQFGTINNFRLGRLPSVPVEWNEINAAWGQTV  311
             Q+ Y Q+QL+KLKKTNVF ATFHI  SGQFG INNFRLGRLPS PV+W+EINAAWGQTV
Sbjct  227  CQLTYTQSQLEKLKKTNVFKATFHISDSGQFGIINNFRLGRLPSAPVDWSEINAAWGQTV  286

Query  312  LLLHALANKMGLKFQRYRLVPYGNHSYLESLTDKSKELPLYCSGGLRFFWDNKFDHAMVA  371
            LLL +LA K+    FQRY+LVPYGNHSY+E L D+ K LPLY SGG RF WD KFD AMVA
Sbjct  287  LLLASLARKISFNFQRYKLVPYGNHSYIEVLEDQ-KVLPLYGSGGFRFLWDTKFDAAMVA  345

Query  372  FLDCVQQFKEEVEKGETRFCLPYRMDVEKGKIEDTGGSGGSYSIKTQFNSEEQWTKALKF  431
            FLDC+QQFKE+VEKG T FCLPYR+D  KGKIEDT      +YSIK QFNSEE WTKALK+
Sbjct  346  FLDCLQQFKEQVEKGNTQFCLPYRID--KGKIEDTASPPHAYSIKIQFNSEEHWTKALKY  403

Query  432  MLTNLKWGLAWVSSQF  447
            MLTNLKW L W+SSQF
Sbjct  404  MLTNLKWALTWISSQF  419
```

Note: The red boxes in the figure above are the predicted antigen epitopes, and the predicted URL is http://imed.med.ucm.es/Tools/antigenic.pl.

5. Amino acid sequence alignment of p62 protein between *Homo Sapiens* and *Spodoptera frugiperda* (Query: *Homo Sapiens*; Sbjct: *Spodoptera frugiperda*).

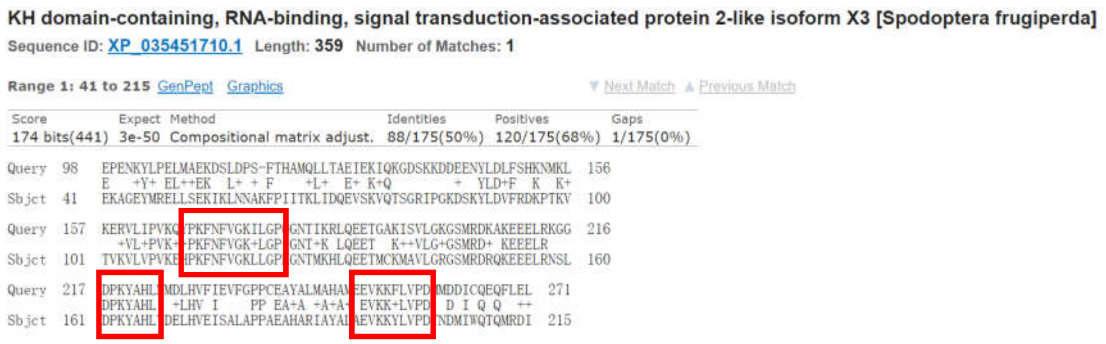

KH domain-containing, RNA-binding, signal transduction-associated protein 2-like isoform X3 [Spodoptera frugiperda]

Sequence ID: XP_035451710.1 Length: 359 Number of Matches: 1

Range 1: 41 to 215 GenPept Graphics ▼ Next Match ▲ Previous Match

Score	Expect	Method	Identities	Positives	Gaps
174 bits(441)	3e-50	Compositional matrix adjust.	88/175(50%)	120/175(68%)	1/175(0%)

```
Query  98   EPENKYLPELMAEKDSLDPS-FTHAMQLLTAEIEKIQKGDSKKDDEENYLDLFSHKNMKL  156
            E   +Y+ EL++EK  L+ + F    +L+  E+ K+Q         +  YLD+F  K  K+
Sbjct  41   EKAGEYMRELLSEKIKLNNAKFPIITKLIDQEVSKVQTSGRIPGKDSKYLDVFRDKPTKV  100

Query  157  KERVLIPVKQ[illegible]PKFNFVGKILGP[illegible]GNTIKRLQEETGAKISVLGKGSMRDKAKEEELRKGG  216
              +VL+PVK+[illegible]PKFNFVGK+LGP GNT+K LQEET  K++VLG+GSMRD+ KEEELR
Sbjct  101  TVKVLVPVKE[illegible]PKFNFVGKLLGP[illegible]GNTMKHLQEETMCKMAVLGRGSMRDRQKEEELRNSL  160

Query  217  DPKYAHL[illegible]MDLHVFIEVFGPPCEAYALMAHA[illegible]EEVKKFLVPD[illegible]MDDICQEQFLEL  271
            DPKYAHL  +LHV I    PP EA+A +A+A+ EVKK+LVPD  D I Q Q  ++
Sbjct  161  DPKYAHL[illegible]DELHVEISALAPPAEAHARIAYAL[illegible]AEVKKYLVPD[illegible]NDMIWQTQMRDI  215
```

Note: The red boxes in the figure above are the predicted antigen epitopes, and the predicted URL is http://imed.med.ucm.es/Tools/antigenic.pl.

6. Amino acid sequence alignment of LC3 (Atg8) protein between *Homo Sapiens* and *Spodoptera frugiperda* (Query: *Homo Sapiens*; Sbjct: *Spodoptera frugiperda*).

autophagy-related protein 8-like [Spodoptera frugiperda]

Sequence ID: XP_035453424.1 Length: 249 Number of Matches: 2

Range 1: 21 to 136 GenPept Graphics ▼ Next Match ▲ Previous Match

Score	Expect	Method	Identities	Positives	Gaps
125 bits(313)	2e-36	Compositional matrix adjust.	56/116(48%)	81/116(69%)	0/116(0%)

```
Query  10   CGKAAVDPADRCKEVQQIRDQHPSKIPVIIERYKGEKQLPVLDKTKFLVPDHVNMSELVK  69
            C K+       R +EV   I+ + P+KIP+I+ERY  E+ LP LDK+KFLVP+ + MS+ +
Sbjct  21   CFKSKKPFISRKEEVMAIKSK[illegible]PTKIPLIVERYHKERNLPTLDKSKFLVPFDITMSQFLV  80

Query  70   IIRRRLQLNPTQAFFLLVNQHSMVSVSTPIADIYEQEKDEDGFLYMVYASQETFGF  125
            IIR R+++ P QA +L++N  SM+S+S  +A  Y+    DEDGFLY+ YASQE FG+
Sbjct  81   IIRNRIRIKPNQALYLIINNKSMLSMSLTMAQAYDNYGDEDGFLYITYASQEVFGY  136
```

Note: The red boxes in the figure above are the predicted antigen epitopes, and the predicted URL is http://imed.med.ucm.es/Tools/antigenic.pl.

7. Amino acid sequence alignment of mTOR protein between *Homo Sapiens* and *Spodoptera frugiperda* (Query: *Homo Sapiens*; Sbjct: *Spodoptera frugiperda*).

serine/threonine-protein kinase mTOR-like [Spodoptera frugiperda]

Sequence ID: XP_035443671.1 Length: 2428 Number of Matches: 1

Range 1: 8 to 2428 GenPept Graphics ▼ Next Match ▲ Previous Match

Score	Expect	Method	Identities	Positives	Gaps
2809 bits(7282)	0.0	Compositional matrix adjust.	1435/2544(56%)	1816/2544(71%)	142/2544(5%)

```
Query  25    FASGLKSRNEETRAKAAKELQHYVTMELREMSQEESTRFYDQLNHHIFELVSSSDANERK  84
             F +GLKSRN + + K A+EL H+   ELRE+ QEE T+F D+ NH IFE+VSS+D +E+K
Sbjct  8     FVAGLKSRNVDVQIKTARELYHFAKTELREVPQEELTQFLDEFNHQIFEMVSSNDVHEKK  67

Query  85    GGILAIASLIGVEGGNA-TRIGRFANYLRNLLPSNDPVVMEMASKAIGRLAMAGDTFTAE  143
             GG+LAI  LIG +     TRI RFANYLRNLLPS+D  VME+A+K +GRLA       AE
Sbjct  68    GGVLAIVCLIGGDCDTTKTRITRFANYLRNLLPSSDVGVMELAAKTVGRLATVSGVKRAE  127

Query  144   YVEFEVKRALEWLGADRNEGRRHAAVLVLRELAISVPTFFFQQVQPFFDNIFVAVWDPKQ  203
             YVEFEVKRA EWL  +RNE RRH+AVL+L+ELA ++PT+F+QQV  FFD+I VA+ DPK
Sbjct  128   YVEFEVKRAFEWLSEERNEGRRHSAVLLLKELA[illegible]AMPTYFYQQVSGFFDHILVALKDPKP  187

Query  204   AIREGAVAALRACLILTTQRE--PKEMQKPQWYRHTFEEAEKGFDETLAKEKGMNRDDRI  261
              IRE A  ALRA L++T QRE   +   KPQWY   +EEA   FDE  AKEKG+ ++DR+
Sbjct  188   QIREAAAKALRAGLVVTAQRETAKQSTAKPQWYMQCYEEAMLSFDEIPAKEKGITKEDRV  247

Query  262   HGALLILNELVRISSMEGERLREE-MEEITQQQLVHDKYCKDLMGFGTKPRHITPFTSFQ  320
             HG LLILNEL+R S+   E+      M+ +  Q+ +         D++   TK
Sbjct  248   HGGLLILNELLRCSNAVWEKKYSYLMQSLDSQKDI--TVSDDIIFISTK-----------  294

Query  321   AVQPQQSNALVGLLGYSSHQGLM--GFGTSPSPAKSTLVESRCCRDLMEEKFDQVCQWVL  378
                         L   S  +G +  GF T          + ES  CR L+ ++F+++C+ V+
Sbjct  295   ------------LHSPSVKRGYLCDGFKTENVTYPVPIYESEVCRRLLTKRFERICKDVM  342

Query  379   KCRNSKNSLIQMTILNLLPRLAAFRPSAFTDTQYLQDTMNHVLSCVK-KEKERTAAFQAL  437
               R  K    +    ++PRLAAF    F + +YL  TM+++LSC++ +EK+R  AF  L
Sbjct  343   NQRFLKTQGVPQILFVIIPRLAAFNKEFFLN-KYLTSTMSYLLSCLRSREKDRNMAFTTL  401

Query  438   GLLSVAVRSEFKVYLPRVLDIIRAALPPKDFAHKRQKAMQVDATVFTCISMLARAMGPGI  497
             GL++ A+ ++ K Y+P ++D+I+  LP +D  K  K + +D ++F CI++L  A+    +
Sbjct  402   GLMAAAIENDIKNYIPCIMDVIKQMLPVRDTQTK--KRIWIDPSIFACITLLGSAVKDLV  459

Query  498   QQDIKELLEPMLAVGLSPALTAVLYDLSRQIPQLKKDIQDGLLKMLSLVLMHKPLRHPGM  557
               DIKELL+ M A GLSP+LT  L +LS  IP L+ +I +GLL MLSLVL +KP  HPG+
Sbjct  460   TTDIKELLDAMFATGLSPSLTICLRELSNNIPSLRAEISEGLLNMLSLVLRNKPFLHPGV  519

Query  558   PKGLAHQLASPGLTTLPEASDVGSITLALRTLGSFEFEG-HSLTQFVRHCADHFLNSEHK  616
             P+ L  Q+++  L   P+  D  SI LALRTLGSF FEG HSL  FVR CADHFL SE +
Sbjct  520   PRSLEQQMSNMSLVMEPQ--DTASITLALRTLGSFNFEGCHSLLTFVRRCADHFLQSEQQ  577

Query  617   EIRMEAARTCSRLLTPSIHLISGHAHVVSQTAVQVVADVLSKLLVVGITDPDPDIRYCVL  676
             EIR+EA +T ++LL  S+            S+T   + A+V+ KLLVV +TDPD ++RY VL
Sbjct  578   EIRLEAVKTAAKLLADSM---IRTMKTPSRTLTLLTAEVVGKLLVVTVTDPDYEVRYWVL  634

Query  677   ASLDERFDAHLAQAENLQALFVALNDQVFEIRELAICTVGRLSSMNPAFVMPFLRKMLIQ  736
              SL+  FD HLAQ ENL  LF+A+ND+   IRELAICTVGRLS +NPA+VMP LRK LIQ
Sbjct  635   ESLNNMFDIHLAQIENLSFLFMAMNDEHLAIRELAICTVGRLSVVNPAYVMPGLRKTLIQ  694

Query  737   ILTELEHSGIGRIKEQSARMLGHLVSNAPRLIRPYMEPILKALILKLKDPDPDPNPGVIN  796
              LTELEHSG+ R KEQ+ARML +L+ +AP+LI+PYME IL  L+ KL++ D   NPGV+
Sbjct  695   FLTELEHSGMSRNKEQAARMLDNLIRHAPKLIKPYMETILNVLVPKLRESDS--NPGVVI  752

Query  797   NVLATIGELAQVSGLE--MRKWVDELFIIIMDMLQDSSLLAKRQVALWTLGQLVASTGYV  854
             +VL  IG LA V G    ++KW+ +L  I++++L D++   KR VALW  GQL+ +TG+V
Sbjct  753   SVLKAIGHLADVHGDNSGLKKWLPDLLSILLELLSDATATEKRSVALWAFGQLIGATGHV  812

Query  855   VEPYRKYPTLLEVLLNFLKTEQNQGTRREAIRVLGLLGALDPYKHKVNIGMIDQSRDASA  914
             V PY +YPTL++VLLNFLKTEQ    RRE IRVLGLLGALDPYKHK+  G+I +  D+S
Sbjct  813   VTPYTEYPTLMDVLLNFLKTEQQPKDRRETIRVLGLLGALDPYKHKITRGLIGEKPDSSL  872

Query  915   VSLSESKSSQDSSDYSTSEMLVNMGNLPLDEFYPAVSMVALMRIFRDQSLSHHHTMVVQA  974
             V +++SK+ +++ D +TSEMLVNM +  LDE+YPA+ +  LMRI RD +L  HHT VVQA
Sbjct  873   VPVADSKTEENNFDMTTSEMLVNMSSTVLDEYYPAIVISTLMRILRDPTLQQHHTSVVQA  932

Query  975   ITFIFKSLGLKCVQFLPQVMPTFLNVIRVCDG-AIREFLFQQLGMLVSFVKSHIRPYMDE  1033
             +TFIF+SLG+KCV ++ +V P+ L V R  D    REFLF QL  L++ VK HIR Y+D+
Sbjct  933   VTFIFQSLGIKCVPYISRVTPSLLYVARATDNNTFREFLFTQLARLIAIVKQHIRNYLDK  992

Query  1034  IVTLMREFWVMNTSIQSTIILLIEQIVVALGGEFKLYLPQLIPHMLRVFMHDNSPGRIVS  1093
             I  L+REFW  N+S+Q T+ILL+E I VALG EFK+YLPQL+P +LRV  HD S  RIV+
Sbjct  993   IFDLIREFWTPNSSLQPTLILLVEYIAVALGSEFKVYLPQLMPQVLRVLAHDTSKDRIVT  1052

Query  1094  IKLLAAIQLFGANLDDYLHLLLPPIVKLFDAPEAPLPSRKAALETVDRLTESLDFTDYAS  1153
              KLL A+Q F  NLDDY+HL++P IVKLFDA + P+   K A+ETVD L+++L++ +  S
Sbjct  1053  QKLLYALQKFEDNLDDYMHLVIPSIVKLFDATDCPIDVAKVAMETVDYLSDTLNYGELIS  1112

Query  1154  RIIHPIVRTLDQSPELRSTAMDTLSSLVFQLGKKYQIFIPMVNKVLVRHRINHQRYDVLI  1213
             RIIHP+VR+LD   +LR TAMDTL +++ Q G+K+  FIP+V KV+V+H+I HQ Y++L+
Sbjct  1113  RIIHPLVRSLDTCAQLRPTAMDTLCAIIVQQGRKFTDFIPLVQKVVVKHKIQHQNYELLL  1172

Query  1214  CRIVKGYTLADEEEDPLIYQHRMLRSGQGDALASGPVETG-PMKKLHVSTINLQKAWGAA  1272
              R+      LA +E   L     R  R+   +A        +T   ++KL V+  NL+ AW  +
Sbjct  1173  SRMQSSQNLAMDEF--LQSTRRRPRNNNQEARIGYTCDTSQSIRKLCVNAHNLKLAWTVS  1230

Query  1273  RRVSKDDWLEWLRRLSLELLKDSSSPSLRSCWALAQAYNPMARDLFNAAFVSCWSELNED  1332
              RVSKDDWLEWLRR S+ LL +S SP+LR+C ALA  Y+ + RDLFNAAFVSCW+EL++
Sbjct  1231  SRVSKDDWLEWLRRFSIGLLTESHSPALRACLALAHNYSQLLRDLFNAAFVSCWTELDKT  1290

Query  1333  QQDELIRSIELALTSQDIAEVTQTLLNLAEFMEHSDKGPLPLRDDNGIVLLGERAAKCRA  1392
              + EL  ++E ALT+ D  E+  T+LNLAEFMEH + G LP+        LLGERA  CRA
Sbjct  1291  SRQELSNALEQALTAPDAPELAHTVLNLAEFMEHCEGGALPISTH----LLGERAMHCRA  1346
```

```
Query  1393  YAKALHYKELEFQKGPTPAILESLISINNKLQQPEAAAGVLEYAM--KHFGE--LEIQAT  1448
             YAKALHYKE EF+ G T  ++E+LI INNKLQQ EAA G+LE  M  +  G+  L++Q
Sbjct  1347  YAKALHYKEEEFRNGATSQVVEALIHINNKLQQKEAAEGLLERVMAQREAGDTSLKVQIR  1406

Query  1449  WYEKLHEWEDALVAYDKKMDTNKDDPELMLGRMRCLEALGEWGQLHQQCCEKWTLVNDET  1508
             WYEKLH WE AL  Y +K++ +  D E  LG +RC EALGEW +L+     +KW  + +E
Sbjct  1407  WYEKLHNWEKALDLYGEKLNVDMGDMESYLGELRCFEALGEWVELYNTVSKKWVKMTNEE  1466

Query  1509  QAKMARMAAAAAWGLGQWDSMEEYTCMIPRDTHDGAFYRAVLALHQDLFSLAQQCIDKAR  1568
             + K AR+AAA+AWGL +WDSM +Y   +P +T DGAFYRAVL +H  F L++Q ID+AR
Sbjct  1467  KCKAARLAAASAWGLNEWDSMAKYVRFLPENTQDGAFYRAVLNIHNGEFELSKQYIDQAR  1526

Query  1569  DLLDAELTAMAGESYSRAYGAMVSCHMLSELEEVIQYKLVPERREIIRQIWWERLQGCQR  1628
              LLD+ELTA+AGESY RAYGA+V+  +L+ELEEVI YK V ERRE IRQ WW RLQG QR
Sbjct  1527  TLLDSELTAVAGESYQRAYGALVNAQLLAELEEVITYKSVVERRESIRQAWWTRLQGGQR  1586

Query  1629  IVEDWQKILMVRSLVVSPHEDMRTWLKYASLCGKSGRLALAHKTLVLLLGVDPSRQLDHP  1688
             +VEDW++IL VRSLV++P EDM TWLK+ASLC KSG    AHKTLV+LLG DPS+  D P
Sbjct  1587  LVEDWRRILQVRSLVLTPQEDMATWLKFASLCRKSGAPRQAHKTLVMLLGTDPSKNKDMP  1646

Query  1689  LPTVHPQVTYAYMKNMWKSARKIDAFQHMQHFVQTMQQQAQHAIATEDQQHKQELHKLMA  1748
             LPT  P++T AY K++W +  K  A+  +Q +V + +        T D +H     +L+A
Sbjct  1647  LPTHEPRLTLAYAKHLWVAGDKQLAYDQLQRYVDSTE--------TGDAEH----CRLLA  1694

Query  1749  RCFLKLGEWQLNLQGINESTIPKVLQYYSAATEHDRSWYKAWHAWAVMNFEAVLHYKHQN  1808
             RC LKLG W  +L GIN+ +IP++L+ Y+AAT+    WYKAWHAWA MNFE VL YKHQ+
Sbjct  1695  RCHLKLGSWCESLLGINKLSIPEILRNYAAATDLSSDWYKAWHAWAYMNFETVLFYKHQD  1754

Query  1809  QARDEKKKLRHASGANITNATTAATTAATATTTASTEGSNSESEAESTENSPTPSPLQKK  1868
                                                A+  G  SE         P P  +Q+
Sbjct  1755  ---------------------------------ANAGGGQSERR-------PLPECIQQ-  1773

Query  1869  VTEDLSKTLLMYTVPAVQGFFRSISLSRGNNLQDTLRVLTLWFDYGHWPDVNEALVEGVK  1928
                        +TVPAV+GFF+SI LS G++LQDTLR+LTLWFDYGH+P V+EALVEG++
Sbjct  1774  -----------HTVPAVEGFFKSIKLSHGSSLQDTLRLLTLWFDYGHYPAVHEALVEGIR  1822

Query  1929  AIQIDTWLQVIPQLIARIDTPRPLVGRLIHQLLTDIGRYHPQALIYPLTVASKSTTTARH  1988
              I+I+ WLQVIPQLIARIDTPR LVG+LIH LL DIG+ HPQAL+YPLTVASKS+  AR
Sbjct  1823  TIEINVWLQVIPQLIARIDTPRALVGKLIHSLLIDIGKSHPQALVYPLTVASKSSFIARK  1882

Query  1989  NAANKILKNMCEHSNTLVQQAMMVSEELIRVAILWHEMWHEGLEEASRLYFGERNVKGMF  2048
             NAAN+ILK+MC HS+ LV QA M+SEELIRVAILWHE WHE LEEASRLYF E +V  MF
Sbjct  1883  NAANQILKSMCTHSSNLVNQAAMISEELIRVAILWHEQWHEALEEASRLYFSENDVNAMF  1942

Query  2049  EVLEPLHAMMERGPQTLKETSFNQAYGRDLMEAQEWCRKYMKSGNVKDLTQAWDLYYHVF  2108
             + LEPLHAM+ERGPQTLKE SF QAYGRDL EAQEWC +Y +SG V+DL+QAWDLYYHVF
Sbjct  1943  KTLEPLHAMLERGPQTLKEVSFTQAYGRDLNEAQEWCNRYKESGQVRDLSQAWDLYYHVF  2002

Query  2109  RRISKQLPQLTSLELQYVSPKLLMCRDLELAVPGTYDPNQPIIRIQSIAPSLQVITSKQR  2168
             RRIS+QLPQLTSLELQYVSP+LL CRDLELAVPG+Y P+Q +IRI +I  SLQVITSKQR
Sbjct  2003  RRISRQLPQLTSLELQYVSPRLLNCRDLELAVPGSYVPDQDLIRIANIQSSLQVITSKQR  2062

Query  2169  PRKLTLMGSNGHEFVFLLKGHEDLRQDERVMQLFGLVNTLLANDPTSLRKNLSIQRYAVI  2228
             PR+L + GSNG +++FLLKGHEDLRQDERVMQLFGLVNTLL  DP + R++L+IQRYAVI
Sbjct  2063  PRRLCIRGSNGKDYIFLLKGHEDLRQDERVMQLFGLVNTLLQADPDTFRRDLAIQRYAVI  2122

Query  2229  PLSTNSGLIGWVPHCDTLHALIRDYREKKKILLNIEHRIMLRMAPDYDHLTLMQKVEVFE  2288
             PLSTNSGLIGWVPHCDTLH+LIRDYREK+K LLNIEHRIM RMA D D L LMQKVEVFE
Sbjct  2123  PLSTNSGLIGWVPHCDTLHSLIRDYREKRKCLLNIEHRIMQRMASDLDKLMLMQKVEVFE  2182

Query  2289  HAVNNTAGDDLAKLLWLKSPSSEVWFDRRTNYTRSLAVMSMVGYILGLGDRHPSNLMLDR  2348
             HA+ +TAGDDLAKLLWLKSPSSEVWF+RRTNYTRSLAVMSMVGYILGLGDRHPSN+MLDR
Sbjct  2183  HALEHTAGDDLAKLLWLKSPSSEVWFERRTNYTRSLAVMSMVGYILGLGDRHPSNIMLDR  2242

Query  2349  LSGKILHIDFGDCFEVAMTREKFPEKIPFRLTRMLTNAMEVTGLDGNYRITCHTVMEVLR  2408
             ++GK LHIDFGDCFEVA+TR+KFPEKIPFRLTRML NAMEVTG++G YR TC +VMEVL
Sbjct  2243  VTGKFLHIDFGDCFEVAVTRDKFPEKIPFRLTRMLINAMEVTGIEGTYRRTCESVMEVLH  2302

Query  2409  EHKDSVMAVLEAFVYDPLLNWRLMDTNTKGNKRSRTRTDSYSAGQSVEILDGVELGEPAH  2468
              HKDSVMAVLEAFVYDPLLNWRL+D         R+R D+     S    D     +P+
Sbjct  2303  RHKDSVMAVLEAFVYDPLLNWRLIDAG------RRSRADAEVCSTS----DPTSSPQPSR  2352

Query  2469  KKTGTTVPESIHSFIGDGLVKPEA--LNKKAIQIINRVRDKLTGRDFSH-DDTLDVPTQV  2525
              +        IH  + D L +P    LNK+A+ I+NRVRDKLTGRDF H D+ + V  QV
Sbjct  2353  NR--------IHIGLNDTLDQPAETNLNKRALAIVNRVRDKLTGRDFPHIDEIVSVQKQV  2404

Query  2526  ELLIKQATSHENLCQCYIGWCPFW  2549
             +LLI+QATS+ENLCQCY+GWCPFW
Sbjct  2405  DLLIQQATSNENLCQCYVGWCPFW  2428
```

Note: The red boxes in the figure above are the predicted antigen epitopes, and the predicted URL is http://imed.med.ucm.es/Tools/antigenic.pl.

8. Amino acid sequence alignment of ULK1 protein between *Homo Sapiens* and *Spodoptera frugiperda* (Query: *Homo Sapiens*; Sbjct: *Spodoptera frugiperda*).

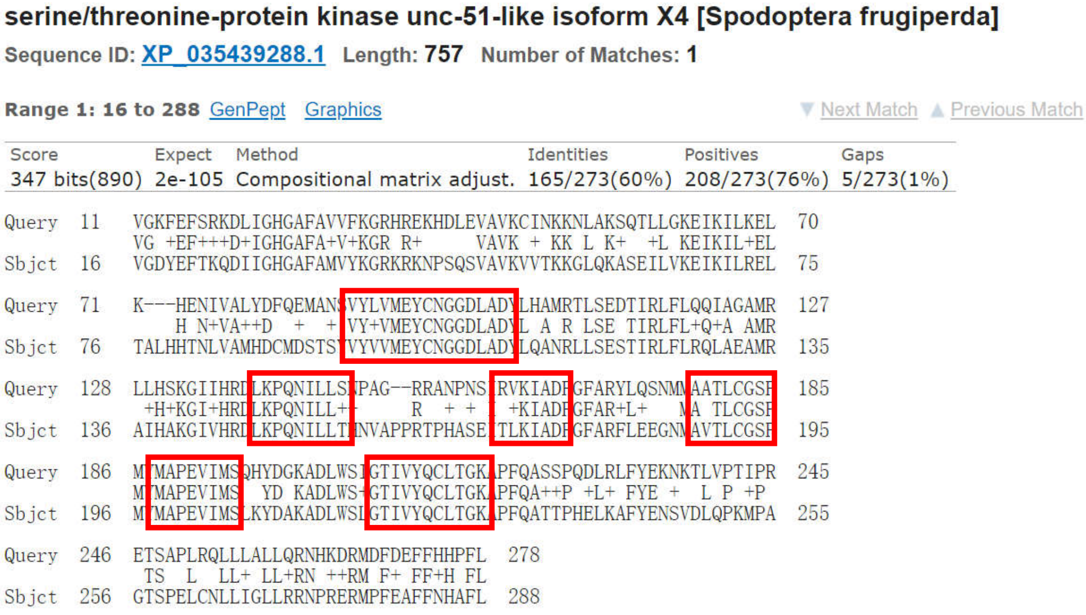

serine/threonine-protein kinase unc-51-like isoform X4 [Spodoptera frugiperda]

Sequence ID: XP_035439288.1 **Length:** 757 **Number of Matches:** 1

Range 1: 16 to 288 GenPept Graphics ▼ Next Match ▲ Previous Match

Score	Expect	Method	Identities	Positives	Gaps
347 bits(890)	2e-105	Compositional matrix adjust.	165/273(60%)	208/273(76%)	5/273(1%)

```
Query  11   VGKFEFSRKDLIGHGAFAVVFKGRHREKHDLEVAVKCINKKNLAKSQTLLGKEIKILKEL  70
            VG +EF+++D+IGHGAFA+V+KGR R+      VAVK + KK L K+  +L KEIKIL+EL
Sbjct  16   VGDYEFTKQDIIGHGAFAMVYKGRKRKNPSQSVAVKVVTKKGLQKASEILVKEIKILREL  75

Query  71   K---HENIVALYDFQEMANSVYLVMEYCNGGDLADYLHAMRTLSEDTIRLFLQQIAGAMR  127
                H N+VA++D  +   + VY+VMEYCNGGDLADYL A R LSE TIRLFL+Q+A AMR
Sbjct  76   TALHHTNLVAMHDCMDSTSYVYVVMEYCNGGDLADYLQANRLLSESTIRLFLRQLAEAMR  135

Query  128  LLHSKGIIHRDLKPQNILLSNPAG--RRANPNSIRVKIADFGFARYLQSNMMAATLCGSP  185
             +H+KGI+HRDLKPQNILL++      R  + + I +KIADFGFAR+L+   MA TLCGSP
Sbjct  136  AIHAKGIVHRDLKPQNILLTHNVAPPRTPHASEITLKIADFGFARFLEEGNMAVTLCGSP  195

Query  186  MYMAPEVIMSQHYDGKADLWSIGTIVYQCLTGKAPFQASSPQDLRLFYEKNKTLVPTIPR  245
            MYMAPEVIMS  YD KADLWS+GTIVYQCLTGKAPFQA++P +L+ FYE +  L P +P
Sbjct  196  MYMAPEVIMSLKYDAKADLWSLGTIVYQCLTGKAPFQATTPHELKAFYENSVDLQPKMPA  255

Query  246  ETSAPLRQLLLALLQRNHKDRMDFDEFFHHPFL  278
             TS  L  LL+ LL+RN ++RM F+ FF+H FL
Sbjct  256  GTSPELCNLLIGLLRRNPRERMPFEAFFNHAFL  288
```

Note: The red boxes in the figure above are the predicted antigen epitopes, and the predicted URL is http://imed.med.ucm.es/Tools/antigenic.pl.

9. Amino acid sequence alignment of p70s6k protein between *Homo Sapiens* and *Spodoptera frugiperda* (Query: *Homo Sapiens*; Sbjct: *Spodoptera frugiperda*).

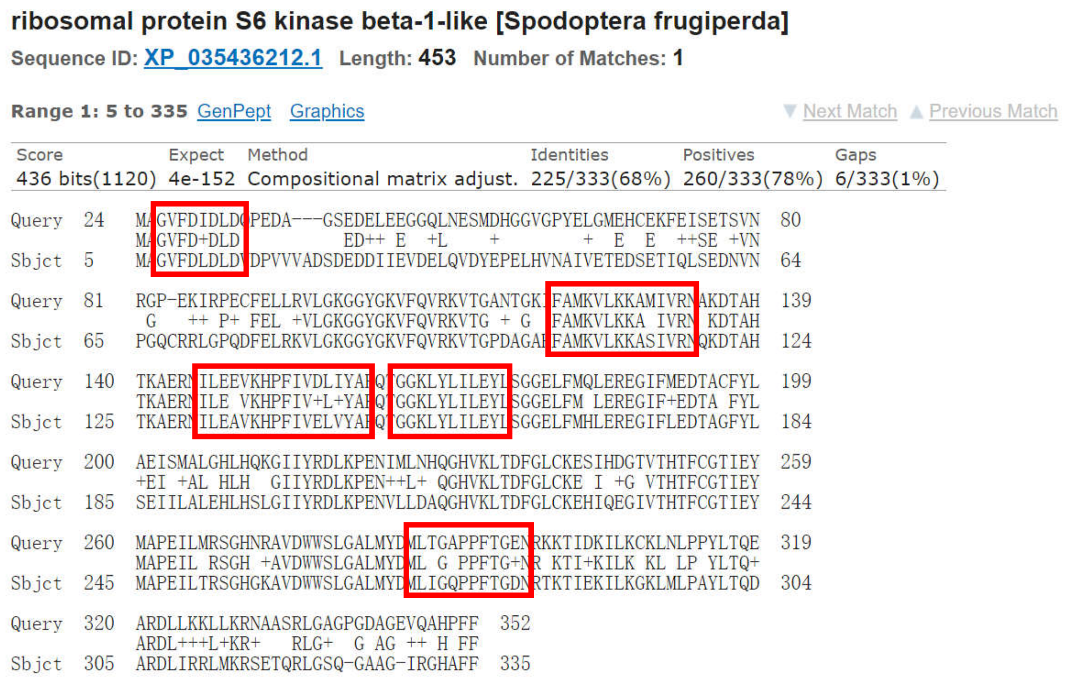

ribosomal protein S6 kinase beta-1-like [Spodoptera frugiperda]

Sequence ID: XP_035436212.1 Length: 453 Number of Matches: 1

Range 1: 5 to 335 GenPept Graphics ▼ Next Match ▲ Previous Match

Score	Expect	Method	Identities	Positives	Gaps
436 bits(1120)	4e-152	Compositional matrix adjust.	225/333(68%)	260/333(78%)	6/333(1%)

```
Query  24   MAGVFDIDLDQPEDA---GSEDELEEGGQLNESMDHGGVGPYELGMEHCEKFEISETSVN  80
            MAGVFD+DLD          ED++ E  +L     +         +  E  E  ++SE +VN
Sbjct  5    MAGVFDLDLDVDPVVVADSDEDDIIEVDELQVDYEPELHVNAIVETEDSETIQLSEDNVN  64

Query  81   RGP-EKIRPECFELLRVLGKGGYGKVFQVRKVTGANTGKIFAMKVLKKAMIVRNAKDTAH  139
             G   ++ P+ FEL +VLGKGGYGKVFQVRKVTG + G  FAMKVLKKA IVRN KDTAH
Sbjct  65   PGQCRRLGPQDFELRKVLGKGGYGKVFQVRKVTGPDAGAFFAMKVLKKASIVRNQKDTAH  124

Query  140  TKAERNILEEVKHPFIVDLIYAFQTGGKLYLILEYLSGGELFMQLEREGIFMEDTACFYL  199
            TKAERNILE VKHPFIV+L+YAFQTGGKLYLILEYLSGGELFM LEREGIF+EDTA FYL
Sbjct  125  TKAERNILEAVKHPFIVELVYAFQTGGKLYLILEYLSGGELFMHLEREGIFLEDTAGFYL  184

Query  200  AEISMALGHLHQKGIIYRDLKPENIMLNHQGHVKLTDFGLCKESIHDGTVTHTFCGTIEY  259
            +EI +AL HLH  GIIYRDLKPEN++L+ QGHVKLTDFGLCKE I +G VTHTFCGTIEY
Sbjct  185  SEIILALEHLHSLGIIYRDLKPENVLLDAQGHVKLTDFGLCKEHIQEGIVTHTFCGTIEY  244

Query  260  MAPEILMRSGHNRAVDWWSLGALMYDMLTGAPPFTGENRKKTIDKILKCKLNLPPYLTQE  319
            MAPEIL RSGH +AVDWWSLGALMYDML G PPFTG+NR KTI+KILK KL LP YLTQ+
Sbjct  245  MAPEILTRSGHGKAVDWWSLGALMYDMLIGQPPFTGDNRTKTIEKILKGKLMLPAYLTQD  304

Query  320  ARDLLKKLLKRNAASRLGAGPGDAGEVQAHPFF  352
            ARDL+++L+KR+   RLG+  G AG ++ H FF
Sbjct  305  ARDLIRRLMKRSETQRLGSQ-GAAG-IRGHAFF  335
```

Note: The red boxes in the figure above are the predicted antigen epitopes, and the predicted URL is http://imed.med.ucm.es/Tools/antigenic.pl.

10. Amino acid sequence alignment of actin protein between *Homo Sapiens* and *Spodoptera frugiperda* (Query: *Homo Sapiens*; Sbjct: *Spodoptera frugiperda*).

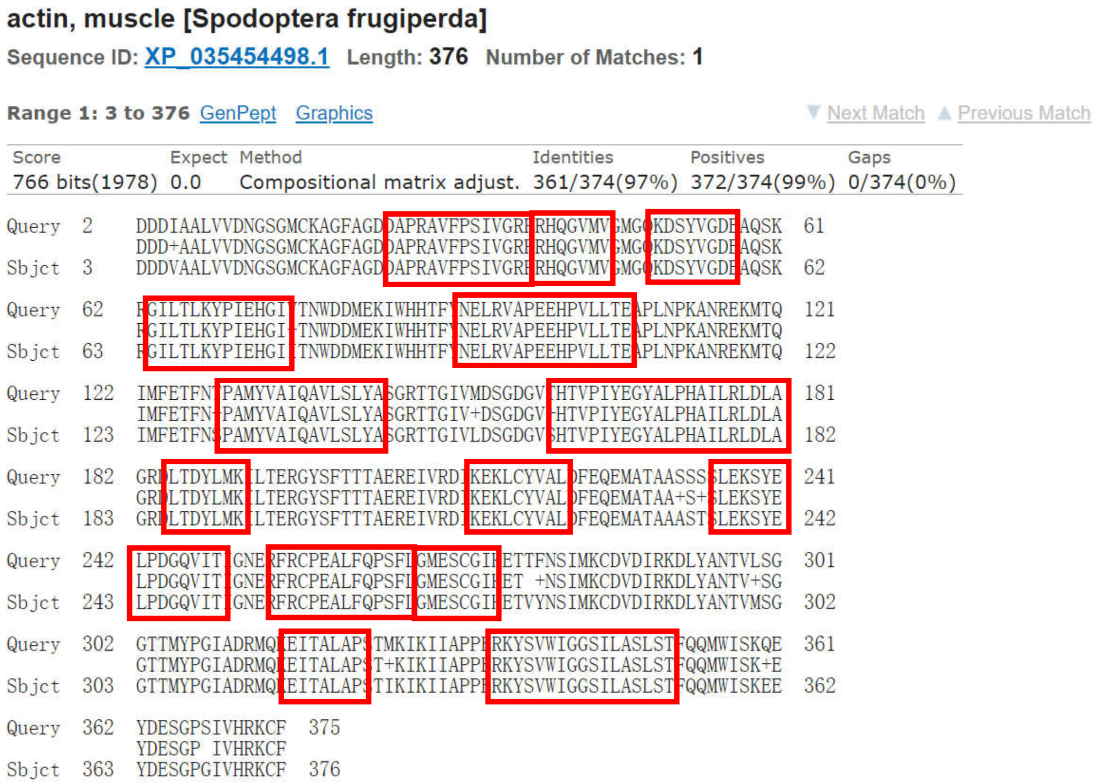

Note: The red boxes in the figure above are the predicted antigen epitopes, and the predicted URL is http://imed.med.ucm.es/Tools/antigenic.pl.
